# Evaluation of histological and ultrastructural changes provoked by prenatal tramadol on postnatal cortical cerebellar neuronal development in rats: possible implication of Ki67, GFAP and MicroRNA-7/P53 signalling trajectories

**DOI:** 10.1007/s10735-024-10189-2

**Published:** 2024-04-19

**Authors:** Walaa Adel Abdelmoez

**Affiliations:** Department of Anatomy and Embryology, Faculty of Medicine, Ain-Shams University, Postal Code: 11591, Abbassia, Cairo Egypt

**Keywords:** Tramadol, Prenatal, Postnatal, MicroRNA-7/P53 Signaling trajectories

## Abstract

Tramadol is a novel centrally acting analgesic. Despite, its implementation during pregnancy may impair neuronal survival and synaptic development in neonatal cerebella. The current investigation assessed the histological and ultrastructural alterations in postnatal cortical cerebellar neuronal development induced by prenatal tramadol. 30 offsprings were divided to **control group I:** fifteen pups born to mothers given saline from D10 till D21 of gestation. **Tramadol-treated group II**: fifteen pups born to mothers received tramadol HCL (50 mg/kg/day) from D10 till D21 of gestation. Pups were categorized into three subgroups (a, b, and c) and offered for sacrifice on the seventh, fourteenth and twenty-first post-natal days. Light microscopic examination revealed the overcrowding and signs of red degeneration affecting purkinje cell layer. Neurodegenerative signs of both purkinje and granule cell neurons were also confirmed by TEM in form of chromatin condensation, dilated Golgi channels, disrupted endoplasmic reticulum, marked infolding of the nuclear envelope and decrease in granule cell precursors. In addition, the astrocytic processes and terminal nerve axons appeared with different degrees of demyelination and decreased number of oligodendrocytes and degenerated mitochondria. Furthermore, group II exhibited an increase in P53 immune expression. The area percentage of apoptotic cells detected by TUNEL assay was significantly increased. Besides to the significant decrease of Ki67 immunoreactivity in the stem neuronal cell progenitors. Quantitative PCR results showed a significant decline in micro RNA7 gene expression in tramadol treated groups resulting in affection of multiple target genes in P53 signaling pathways, improper cortical size and defect in neuronal development.

##  Introduction

Opioids are analgesics utilized to control severe to moderate pain. As a moderate opioid prodrug, tramadol is administered postoperatively to manage acute pain (Wallin et al. 2021). It also acts at µ-opioid receptor by the reuptake inhibition of noradrenaline and serotonin and is a synthetic analog to codeine (Aubry and Carr 2022). Tramadol abuse is on the rise among adolescents who have a prior history of substance addiction and anxiety. A considerable number of adolescent addicts replace other narcotics with tramadol. Tramadol consumption known to be associated with numerous hazards. The most common adverse effects are nausea, disorientation, sedation, dry mouth, sweating and convulsions. Moreover, the drug consumption has been linked to oxidative stress conditions, respiratory distress and other severe adverse effects (Chauhan et al. 2022). The central nervous system and its all subdivisions, particularly the cerebellum, are vulnerable to oxidative stress created by opioids (Liu et al. 2022). Owing to its elevated levels of polyunsaturated fatty acids, high oxygen consumption rate and comparatively low antioxidative enzyme content. Recent years have shown a substantial need for reevaluation of our knowledge regarding some of the most well-known attributes of cerebellar growth, including its origin of its numerous cell types and its geographical distribution (Lowenstein et al. 2023).

Tramadol is generally contraindicated during pregnancy as it has the potential to cause reversible withdrawal symptoms in the fetus. Recent research work has demonstrated that the use of tramadol during pregnancy and lactation have detrimental effects on neuronal development, synapse formation and survival in the cerebrum and cerebellum of the developing progeny (Allen et al. 2021). The potential hazards of tramadol on the cerebellum and the function of opioid receptors in the cerebellum have been depicted in a relatively consistent manner by accumulating evidence (Mohamed et al. 2022). Tramadol has been documented to cause disruptions in the metabolism of various amino acids that are involved in regulating the neuropathophysiology of the cerebellum and to influence the activity of several essential enzymes in the cerebellum. Due to its widespread use and pharmacological properties, tramadol research assessing its effect on malformation risks is urgently required (Simmons et al. 2023).

Conversely, the neonatal cerebellum maintains a considerable capacity for proliferation through the regulation of numerous transcriptional regulators and endogenous microRNA mechanisms (Du et al. 2019). The maturation of the soma and dendritic tree of Purkinje cells (PCs) persists during early postnatal life, concurrent with the proliferation and differentiation of cerebellar neurocytes. However, the precise molecular mechanisms by which neonatal neurocytes undergo maturation and differentiation into cerebellar cells are still unknown (Rahimi-Balaei et al. 2023). MicroRNA-7 (miR-7) is known to have an indispensable regulatory function in the development of tissues and organs. various target molecules are regulated by miR-7, which, according to other studies, is involved in tumorigenesis, ageing, tissue and organ development, among other processes (Chen et al. 2023). The objective of the current research was investigation of the histomorphometric, enzymatic and molecular changes that may occur in the cerebella of offsprings at different phases of development as a result of tramadol administration during pregnancy and highlighting the possible effect of micro RNA7 gene expression alteration on proper cerebellar cortical development in the offsprings.

## Material and methods

### Ethical approval

With approval number FWA 000017585, the Ethical Committee of Ain Shams University granted approval for the investigation. The methods used in this investigation were implemented in adherence to the guidelines set forth by (CARE). The research ethics committee of Ain Shams University, the local medical school.

#### Drugs

The 50 mg capsules of tramadol HCL® were acquired from BERNOPHARM, Sidoarjo – Indonesia. Tramadol hydrochloride was administered at a dose of 50 mg/Kg/day via oral gavage (Faria et al. 2017).

#### Animals

The current investigation utilized thirty adult male and female albino rats, each weighing between 200 and 250 g, from the animal laboratory of the Medical Research Centre (MASRI), Faculty of Medicine, Ain Shams University. Two rats were housed in stainless steel cages, with each cage measuring 30 × 35 cm and accommodating two rats. The study excluded rodents with a disease, those that had been used in previous experiments, those that had difficulty walking, or those with poor fur. The rodents were provided with a light–dark cycle lasting 12 h, unrestricted access to food and water and housing in an environment that met ventilation and environmental standards. Maternal reproduction took place within overnight shelters. The identification of pregnancy was accomplished through the observation of spermatozoa in the vaginal stain on day zero (D0) of gestation. Pregnant rats were divided into two main groups on day seven of pregnancy (D7): group (I) was designated as control group, while group (II) was treated with tramadol. The current study utilized a set of thirty offsprings subsequent to their delivery. The puppy division was as follows:

- **Group I (control groups):** Fifteen puppies were delivered to mothers who were administered saline from day 10 to day 21 of gestation. The young puppies were additionally divided into three subgroups, each consisting of five puppies (Ia, Ib, and Ic). These subgroups were euthanized on the seventh, fourteenth, and twenty-first postnatal days, respectively.
- **Group II (treated groups):** Tramadol HCL (50 mg/kg/day) dissolved in normal saline and administered via oral gavage to fifteen mothers between day 10 and day 21 of gestation was administered (Faria et al. 2017). The critical period of vulnerability in the development of the nervous system (Rice and Barone 2000). The pups were subsequently partitioned into three subgroups (IIa, IIb, and IIc), with each subgroup consisting of five puppies. Sacrifice occurred on the seventh, fourteenth and twenty-first postnatal days, respectively.

Final cerebellar maturation occurred when neuronal morphology and synaptic connectivity completed their development on the twenty-first postnatal day (McKay and Turner 2005). Rats were severely euthanized at the end of the experiment via intraperitoneal xylazine (15 mg/kg) and ketamine (90 mg/kg) in order to be decapitated (Kilic et al. 2021). Subsequently, the cerebella of male albino rats were dissected, promptly extracted and processed for histological and immunohistochemical analysis at various postnatal ages.

### For light microscopic study

#### Histological analysis

Cerebellar tissue samples were fixed in 10% neutral buffered formalin for one day, dehydrated in ascending grades of alcohol, cleared in xylene and embedded in paraffin wax. Five μm serial sections were cut and prepared for the histological (H&E, Nissl stain, modified Bielschowsky’s silver stain) (Ghosh et al. 2021). Other specimens were cleansed for two hours in phosphate buffer before semithin sections were made. Using a glass knife, one-micron-thick sections were carved from glass. Using an Olympus light microscope equipped with an automatic photomicrographic camera system, the pigmented sections were examined. Kodak ASA400 film was used for the color photographs (Bancroft and Gamble 2008)*.*

#### Immunohistochemical analysis of (GFAP) and (Ki-67)

In order to conduct immunostaining, serial sections of 5 μm thickness were transferred from the paraffin blocks to glass slides. The slides were then incubated at 65° C for the duration of the night. The slide sections were deparafinized in xylene for one to two minutes prior to rehydration in ethanol for three minutes per section, followed by distilled water for five minutes. Through a 10-min exposure to 0.3% hydrogen peroxide at room temperature. The activity of endogenous peroxidase was inhibited. Following a rinse in phosphate-buffered saline (PBS), a one-hour incubation in 5% normal goat serum at room temperature effectively inhibited non-specific binding. Anti-GFAP (Kit #MS-280-B0) was utilized to incubate the sections for an approximate volume of 100 µl per section (GFAP, 1:500 dilution) with the primary antibody (Ali and Sonpol 2017) Lab Vision Corporation, Medico Co., Egypt and Anti Ki-67; Kit#PA5-19,462 (rabbit polyclonal antibody, sc-15402, 1:200), ThermoFisher scientific, USA. After washing with PBS for 20 min, the sections were incubated with a secondary biotinylated antibody. A solution containing the enzyme conjugate "Streptavidin–Horseradish peroxidase" was subsequently applied to the sections and left for a duration of 10 min. As a chromogen for secondary antibody binding visualization, 3,3-diaminobenzoic acid (DAB) was dissolved in PBS at a concentration of 0.03% by adding H2O2 immediately prior to use. Hematoxylin is a counterstaining agent (He et al. 2016).

#### Terminal deoxynucleotidyl transferase nick end labeling (TUNEL) assay

In accordance with the protocols provided by the manufacturer, the cortical cerebellar neurons that had experienced apoptosis were identified using a TUNEL assay. The paraffin-embedded tissues were rehydrated with ethanol series (absolute, 95%, 90%, 80%, and 70%, diluted in double distilled water) subsequent to deparaffinization in xylene. The portions were subsequently washed with PBS following this and each subsequent phase. The sections underwent a 30-min incubation period in a damp box set at 37 °C in the absence of light subsequent to a 30-min treatment with proteinase K (20 g/mL in 10 mM Tris/HCI, pH 7.6). Following three five-minute rinses with PBS, 100 L of a DAB substrate was dropwise added to the sections, followed by a 10-min incubation at 25 °C (Duan et al. 2003).

#### P53 immunohistochemistry

Xylene and citrate buffer (pH 6) were used for deparaffinization and retrieval of the antigen at 60 °C respectively. Hydrogen peroxide 3% was used to block the endogenous peroxidase activity. P53 primary antibodies were purchased from ThermoFisher scientific kit #MA5-12,453, USA; then diluted in PBS in dilution 1:1000. The primary antibodies were incubated overnight with tissue sections at 4 °C, then washed. Sections were incubated again for an hour with biotinylated secondary antibodies at room temperature. The immunostaining was identified by using the Avidin–biotin complex (ABC) method (Vectastain Elite ABC kit, Vector Laboratories, Burlingame, CA). Diaminobenzidine (DAB) worked as chromogen. Counterstaining of the slides with hematoxylin, dehydration in graded alcohol, clearance with xylene, and DPX mounting was done (Mousa et al. 2015).

### For electron microscopic study

The processing of cerebellum tissue samples for transmission electron microscopy was carried out in accordance with the established protocol. After fixating fragments of cerebellar tissue for two hours at 4℃ in 2.5% phosphate-buffered glutaraldehyde, they were rinsed with phosphate-buffered saline. The cerebellar specimens were subsequently post-fixed for one hour at 4 °C in a 1% phosphate buffer containing osmium tetroxide. Following dehydration in increasing concentrations of alcohol, the cerebellar samples were submerged in propylene oxide and encapsulated in an epoxy resin mixture. Sections with 1 µm thickness were cut, stained with toluidine blue, and analysed by L/M in order to identify the relevant regions. For the examination of 80-90 nm thick ultrathin sections, lead citrate and uranyl acetate were utilised to generate contrast. The sections were examined using a transmission electron microscope (TEM) located at the Electron Microscopic Unit of Ain Shams University ("Jeol" E.M.-100 CX11; Japan) (Kue 2007).

#### For biochemical analysis

Blood samples were centrifuged at 4,000 round per minute after coagulation for ten minutes. Hypothesis testing was conducted on serum samples to determine the concentration of oxidative stress markers using enzyme-linked immunosorbent assay (ELISA). Following the addition of the attenuated serum sample to the capture antibody-coated ELISA plate, the plate was incubated for a predetermined amount of time. Following the removal of the unbound sample by rinsing, the detection antibody was introduced onto the plate. Substrate was introduced subsequent to incubation and rinsing, absorbance was measured on a spectrophotometer (ELx 800; Bio-Tek Instruments Inc., Winooski, VT, USA) utilizing a microplate reader. The collected serum was analyzed for malondialdehyde dehydrogenase (MDA; Kit# E-BC-K025-S Elabscience Biotechnology, USA) and superoxide dismutase (SOD; Kit#E-BC-K020; Elabscience Biotechnology, USA), markers were quantified in order to assess the oxidative stress state.

## For Estimation of miRNA Expression by Real time PCR (polymerase chain reaction)

### Tissue Homogenization

Tissue was subjected to both disruption and homogenization using the Tissue Ruptor II (Qiagen, Hilden, Germany), a rotor–stator homogenizer. This device disrupts and homogenises single tissue samples simultaneously for 15–90 s, depending on the sample's texture and size, in the presence of lysis buffer. After that, the mixture is centrifuged for 20 min at 4000 rpm. Following this, the cell supernatant is collected in advance of RNA extraction.

### miRNAs extraction & purification

Total mRNA and miRNAs were extracted using **miRNeasy Serum/Plasma Advanced Kit, cat no: 217204** (***Qiagen, Hilden, Germany***) according to the manufacturer’s protocol.

### Reverse transcription

cDNA was synthized by reverse transcription reaction using **mi Script RT Kit** (***Qiagen, Hilden, Germany***).

### miR-7 expression analysis

The quantification of miR-7 gene expression levels was amplified from miRNA extract using a **miScript primer assay primer assays**; [miR-7, cat no: 218300, assay ID: MS00031220]. Primer Evaluation. Utilising miScript Syber green Master mix (Qiagen, Hilden, Germany), the genes for microRNAs were amplified. As a housekeeping gene, the SNORA11 Primer Assay was run. To prepare the PCR reaction mix, 5µL of 2 × miScript Syber green Master mix, 10 × miScript Universal (1µL), and 10 × miScript Hs_miR-7 was added in that order. At ambient temperature (15–25ºC), primer assay (1µL), template cDNA (1µL), and RNase-free water were defrosted. The volume of the reaction was then adjusted to 10 μl per well by adding 2 µL of RNase-free water. Appropriate volumes of the reaction mixture were dispensed into the wells of the Rotor-Disc while the disc was sealed with Rotor-Disc Heat-Sealing Film. The mixture was mixed meticulously yet gingerly. The 5 plex Rotor-Gene PCR Analyzer (Qiagen, Germany) was utilised to process each sample.

As a result, the initial cycle of the real-time cycler was programmed as follows: 15 min of activation at 95 oC to initiate the HotStarTaq DNA Polymerase. Forty cycles of three-step cycling: 15 s of denaturation at 94 oC, 30 s of annealing at 55 oC, and 30 s of extension at 70 oC. Furthermore, the levels of expression were normalised to those of the reference gene SNOR11. In order to normalise the relative expression level (fold change) of miR-Let7a, it was normalised to an internal control (SNORA11) and computed relative to the calibrator (negative control sample) utilising the 2-∆∆Ct test control equation.

## Histomorphometric analysis

### 1-quantitative image analysis

The image analyzer was utilized to conduct the measurements in the Department of Histology and Cell Biology of the Faculty of Medicine at Ain Shams University. The area percentage of immunological staining was computed utilizing seven fields extracted from seven independent serial sections of seven animals in each group. Initially, the image analyzer underwent an automatic calibration process to convert the measurement units (pixels) produced by the application into accurate micrometer units. The regions in each field that exhibited positive responses to anti-GFAP and TUNEL immunostaining were initially enveloped in a blue binary color prior to being quantified within the standard measurement frame. Anti-GFAP and TUNEL expression was quantified by calculating the proportion of positively stained regions to the total area of cortical cerebellar tissue. The thickness of the three cortical layers of the cerebellum was also estimated.

#### 2-statistical analysis

Data were collected, inputted, and analysed on a suitable computer using version 13.0 of the statistical package for the social sciences (SPSS) software for windows. To compare group mean values, a one-way analysis of variance (ANOVA) was implemented. Data for each cohort were presented in the form of the mean and standard deviation (SD). A post hoc Bonferroni test was utilised to ascertain whether any two distinct groups differed in any way. For a value to be considered significant, the P value had to be equal to or less than 0.05. Statistical significance is attributed to results when the P value is 0.05 or less (Sawilowsky 2005).

## Results

### Histopathological results

**Fig. 1:**
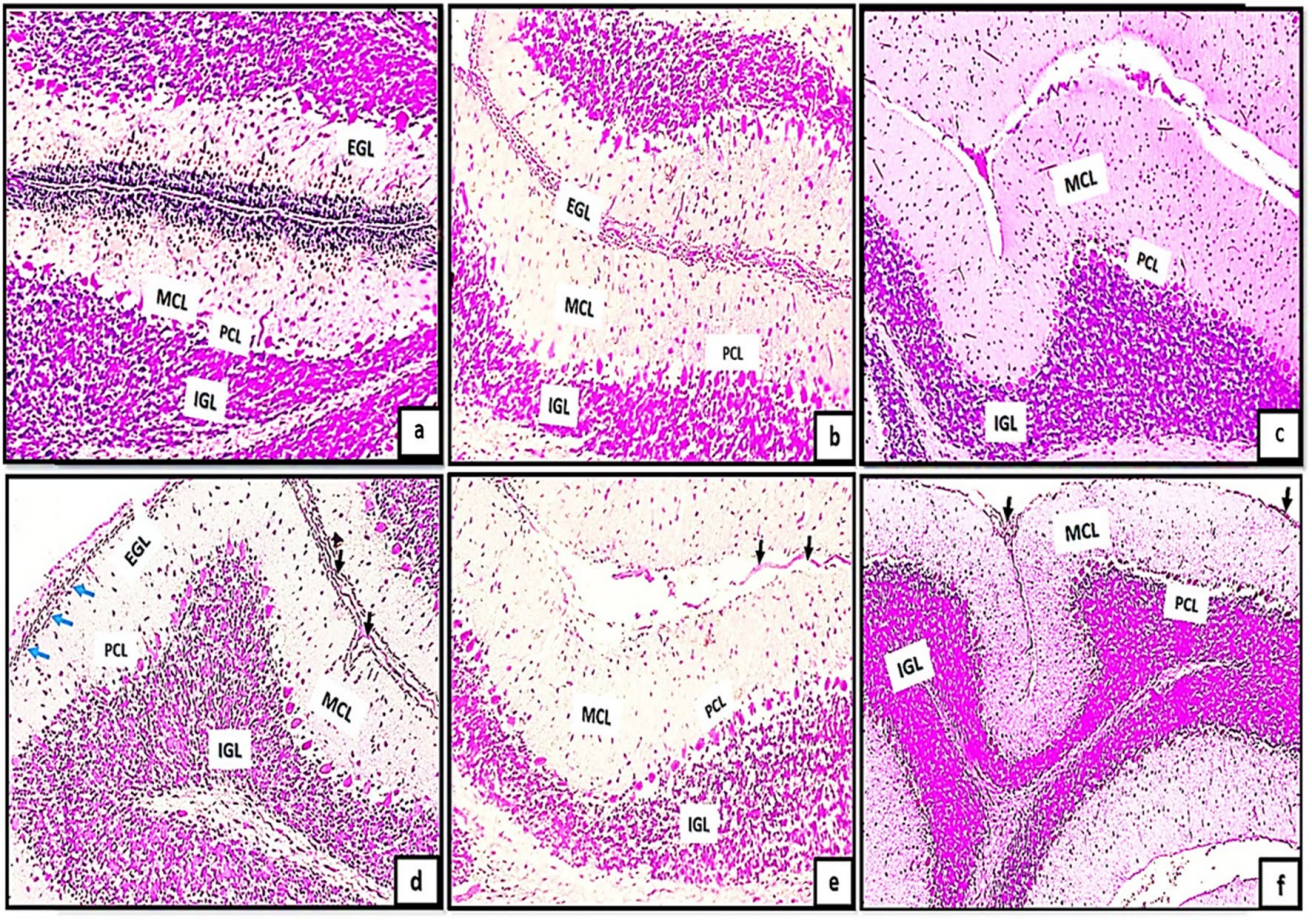

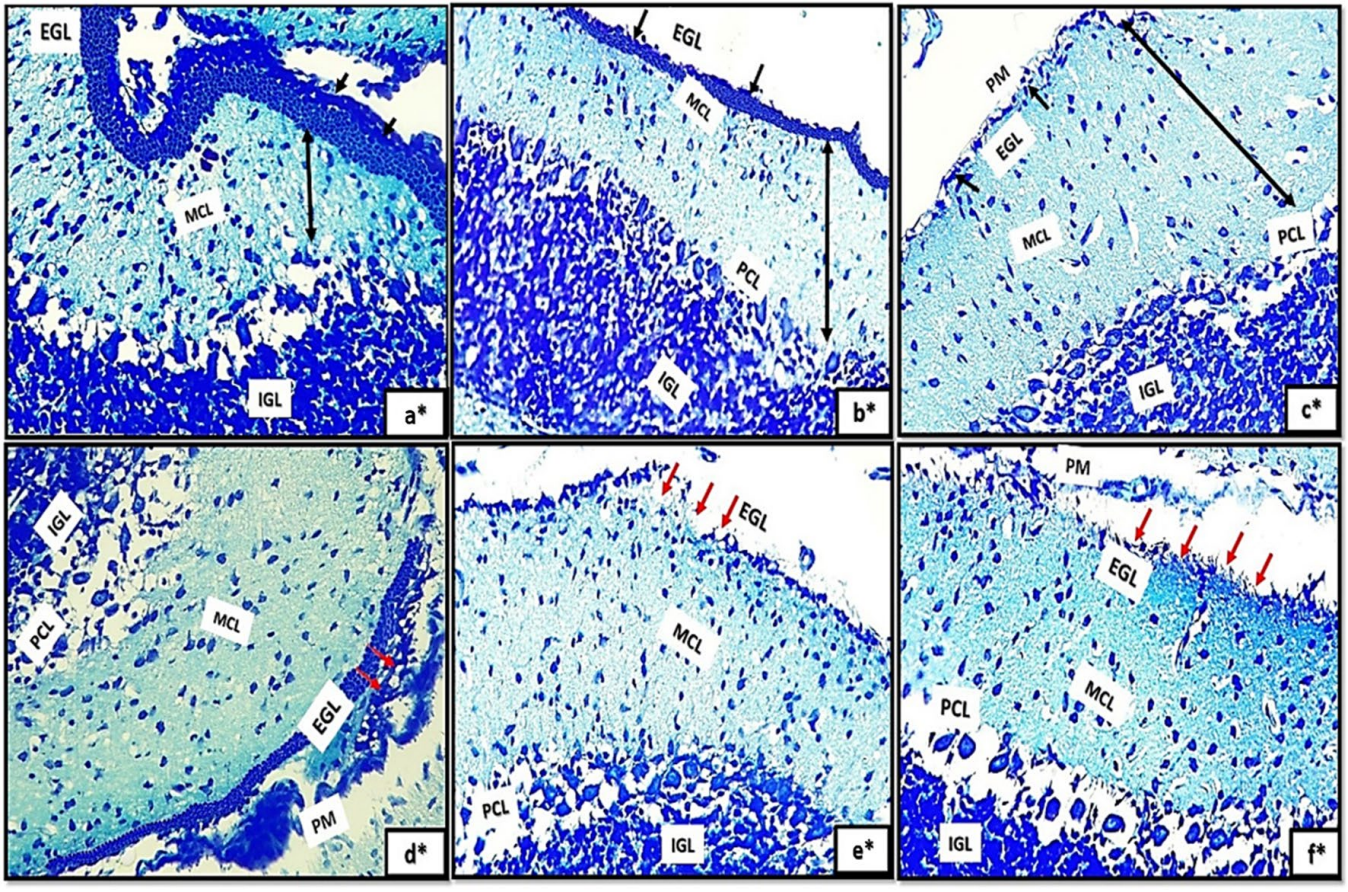
(1a,1b,1c): photomicrographs of transverse sections of albino rat cerebellar cortex from the control groups (Ia, Ib, IC) showing the general architecture of folia of rat cerebellar cortex consisting of external granular cell layer (EGL), molecular cell layer (MCL), purkinje cell layer (PCL) and internal granular cell layer (IGL). Figures (1d,1e,1f): photomicrographs of transverse sections of albino rat cerebellar cortex from tramadol-treated groups (IIa, IIb, IIC) showing the three major cortical cerebellar layers external granular layer (EGL), purkinje cell layer (PCL), internal granular cell layer (IGL). Note, decreased thickness of EGL in (Fig. 1d) and persistence of remnants of external granular layer(EGL) in figs.1e,1f with congested blood vessels are clearly shown in Figs. (1d, 1e, 1f) (black arrows) (H&E,200). Figures (1a*,1b*,1c*): photomicrographs of transverse sections of albino rat cerebellar cortex from the control groups (Ia, Ib, IC) showing the three different layers of rat cerebellar cortex; external granular layer (EGL) appears consisting of multiple layers of granule cells with deeply stained nuclei (Fig. 1a*) and as a very thin continues layer in (Fig. 2b*) and as a remnants of cells on the surface of the cerebellar cortex (Fig. 2c*). note the progressive decrease in its thickness among different control groups (black arrows). molecular cell layer (MCL) composed of multiple neurons of different shapes and sizes (double head black arrow); purkinje cell layer (PCL) composed of one raw of apparently flask shaped cells interposed between both (MCL) and (IGL). The internal granular cell layer (IGL) consists of densely populated granule cells. Figures (1d*,1e*,1f*): photomicrographs of transverse sections of albino rat cerebellar cortex from tramadol treated groups (IIa, IIb, IIC).(Fig. 1d*): showing the cells of EGL with extensive cytoplasmic vacuolations(red arrows) under surface of pia matter(PM); some cells of EGL appears completely degenerated and ruptured to the surface (red arrows) in Fig. 1e*.Remnants of degenerated cells appears under cover of thin rim of pia matter(PM) with irregular shape and rough borders on the surface of the cerebellar cortex (Fig. 1f*). (Nissl stainx400)

Light microscopic examination of H&E of the control groups (Ia, Ib, Ic) revealed the general architecture of folia of rat cerebellar cortex which appeared consisting of external granular cell layer (EGL), molecular cell layer (MCL), purkinje cell layer (PCL) and internal granular cell layer (IGL) Fig. 1a, b, c. The three major cortical cerebellar layers; (EGL), (PCL) and (IGL) and congested dilated blood vessels were also clearly observed in (Fig. 1d, e, f) in tramadol treated groups (IIa, IIb, IIC).

**Fig. 2:**
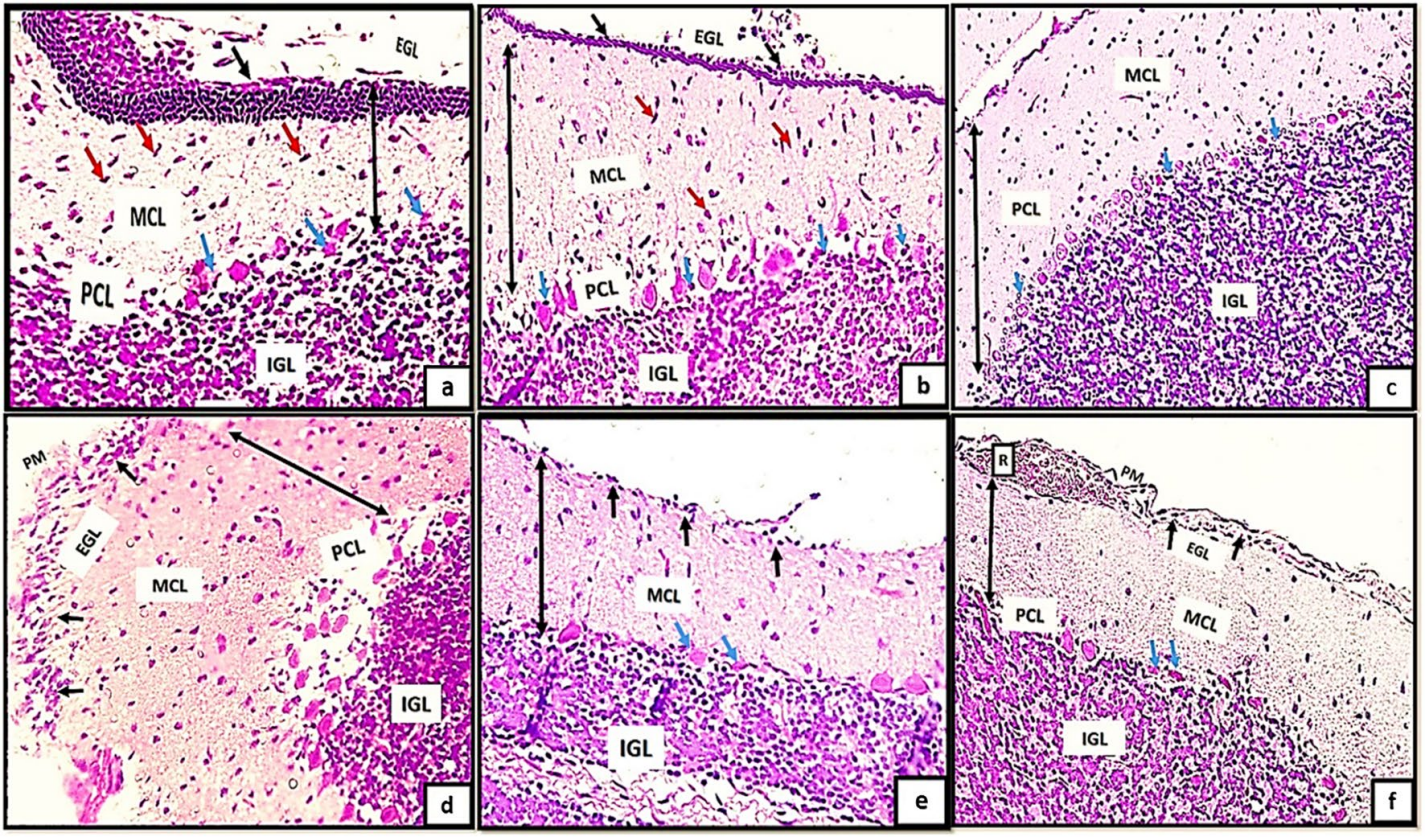

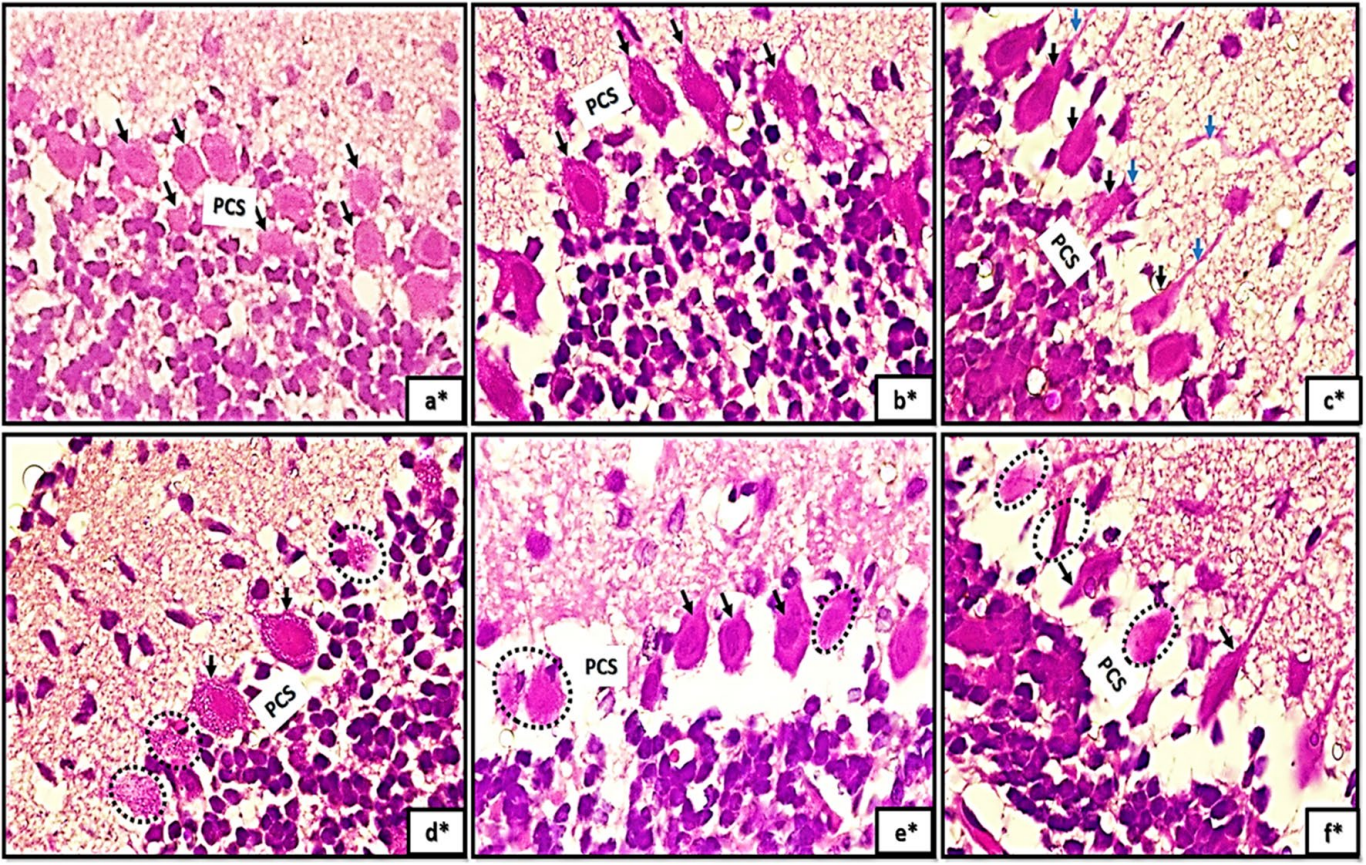
(a,b,c): photomicrographs of transverse section of albino rat cerebellar cortex from the control groups (Ia,Ib,IC) showing the three different layers of rat cerebellar cortex;(EGL),(PCL) and (IGL).Note, the progressive decreased thickness of EGL (Black arrows). The molecular cell layer showing progressive increase in its thickness (double head black arrows), proliferating Bergmann astrocytes clearly shown proximal to purkinje cell layer(PCL)(blue arrows), granule cells are clearly shown migrating through the MCL (red arrows)(figs,2a,2b). Note also the decreased cellularity of the MCL among different control groups. Note also the densely populated granule cells in Fig. 2C.; Figs.(2d,2e,2f): transverse sections of rat cerebellar cortex from tramadol treated groups (IIa, IIb, IIC); showing decreased thickness of EGL under remnants of pia matter (PM) in Fig. 2d (black arrows) and persistence of remnant cells of EGL among all tramadol treated groups (figs.2d,2f) (black arrows); distorted, degenerated purkinje cells (PCL)(blue arrows).Extravasated RBCS clearly observed in (R)(Fig. 2f). Note also the thickness of (MCL) among different tramadol treated groups (double black head arrows).(H&E,400). Figures (2a*,b*,c*): photomicrographs of transverse section of albino rat cerebellar cortex from the control groups (Ia,Ib,IC) showing different degrees of differentiation of purkinje cells(PCS).Fig. 2a*: the purkinje cells appears apparently circular in shape with acidophilic cytoplasm and deeply stained nuclei, they appear crowded and arranged in variables layers (black arrows). Figure 2b*: the purkinje cells begins to attain a pyramidal shape with pointed tapered apex and wide base and becomes arranged in one layer (PCL) (black arrows). Figure 2c*: showing the purkinje cells (PCS) with characteristic flask shape (black arrows); pointed tip and apical dendrites could be observed (blue arrows). (figs,2a*,2b*): Photomicrographs from rat cerebellar cortex groups (2c*,2d*,2f*); showing some healthy purkinje cells (black arrows); others appear distorted degenerated (black dotted encirceled areas). Note that the purkinje cells appears arranged only in group IIa (no signs of mitosis) (H&E,1000)

Furthermore, the transverse sections of rat cerebellar cortex from the control groups (Ia, Ib, IC) stained with Nissl stainning showed the three different layers of rat cerebellar cortex Fig. 1a*, b*, c*. The external granular layer (EGL) appeared consisting of multiple layers of granule cells with deeply stained nuclei (Fig. 1a*) and very thin continues layer in (Fig. 2b*) and as a remnants of cells on the surface of the cerebellar cortex in (Fig. 2c*). The EGL showed progressive decrease in its thickness among different control groups. The molecular cell layer (MCL) consisted of multiple neurons of different shapes and sizes. The purkinje cells (PCs) were arranged in one raw of apparently flask shaped cells interposed between both (MCL) and (IGL). The internal granular cell layer (IGL) consisted of densely populated granule cells. Moreover, cells of EGL exhibited extensive cytoplasmic vacuolations clearly observed in (Fig. 1d*). Some cells of EGL were completely degenerated and ruptured to the surface (Fig. 1e*). Remnants of degenerated cells appears with irregular shape and rough borders on the surface of the cerebellar cortex (Fig. 1f*).

H&E stained sections also revealed the progressive decrease in the thickness of EGL in different control groups. Besides, the molecular cell layer showed decreased cellularity, progressive increase in its thickness. Moreover, proliferating Bergmann astrocytes were clearly shown proximal to purkinje cell layer(PCL). Granule cells were clearly shown migrating through the MCL (Fig. 2a, b) and appeared densely populated in Fig. 2C. Furthermore, the transverse sections of rat cerebellar cortex from tramadol treated groups (IIa, IIb, IIC); the EGL was persistent among all tramadol treated groups (Fig. 2d, f). Decreased thickness of MCL was observed among tramadol treated groups with extravasated RBCS were clearly shown in EGL under the cover of pia matter (Fig. 2f).

With high magnification of H&E stained sections the purkinje cells(PCS) showed different degrees of differentiation among different control groups. PCS appeared apparently circular in shape with acidophilic cytoplasm and deeply stained nuclei, they appeared crowded and arranged in variable layers Fig. 2a*. In group Ia the purkinje cells began to attain a pyramidal shape with pointed tapered apex and wide base and became arranged in just one layer (PCL) Fig. 2b*. The characteristic flask shape of purkinje cells were clearly observed in sections of group IIc; pointed tip and apical dendrites could be clearly observed Fig. 2c*. However, signs of degeneration were clearly observed in sections of groups IIa, IIb, IIc (Fig. 2d*, e*, f*).

**Fig. 3:**
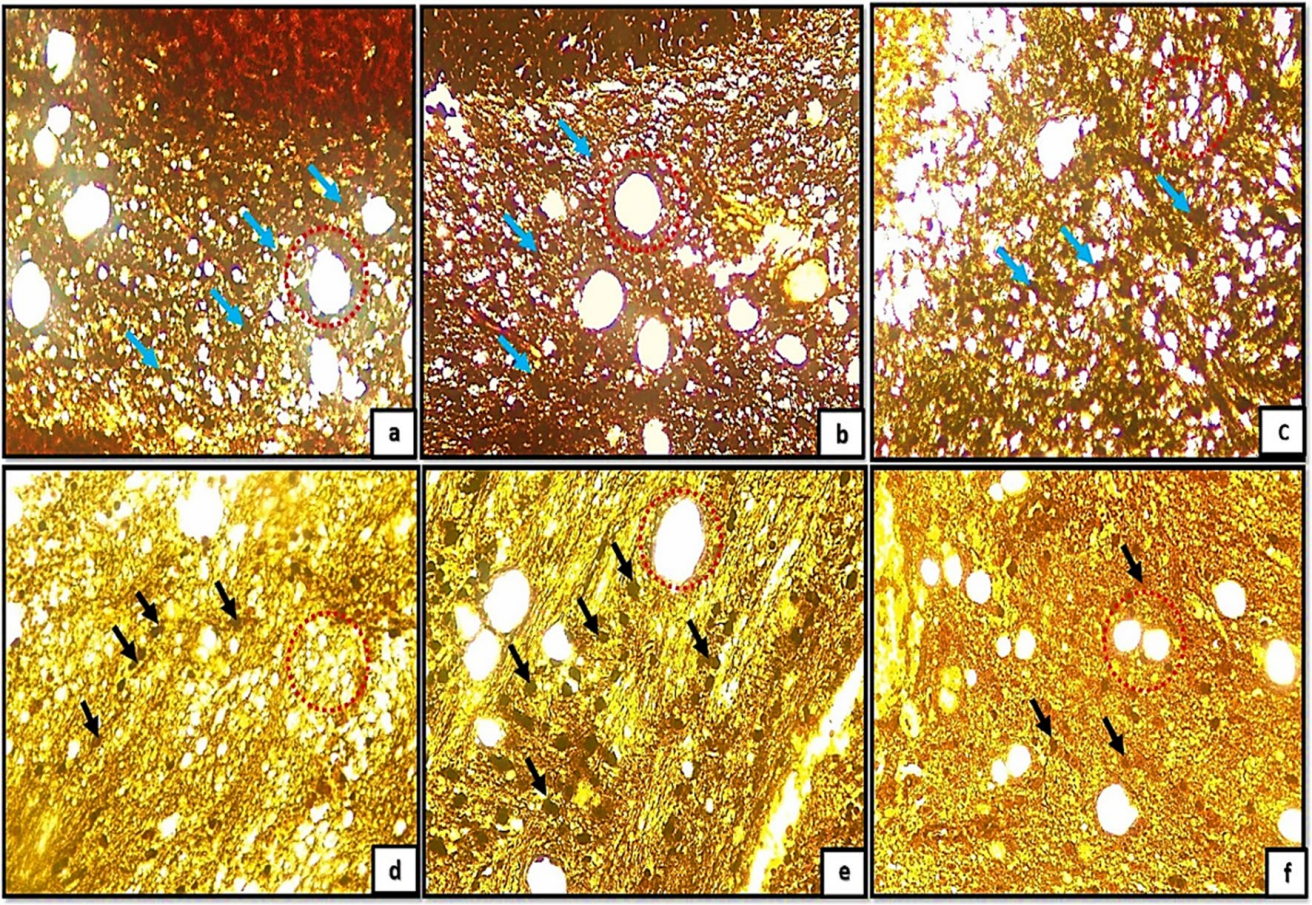
(3a,3b,3c): photomicrographs of transverse sections of albino rat cerebellar cortex from the control groups (Ia, Ib, IC) showing different degrees (minimal, moderate, extensive) of distribution of oligodendroglia (blue arrows) appears as deeply stained circular dots along the terminal nerve axons with different degrees of myelination (dotted red encircled areas); the myelin sheath appears as irregular faint blue line surrounding the terminal nerve axons. Figures (3d,3e,3f) showing transverse sections of rat cerebellar cortex from tramadol treated groups (IIa, IIb, IIc); exhibiting extensive decrease in number of oligodendroglia (black arrows) and extensive demyelination of the terminal nerve axons (dotted red encirceled areas).(Modified Bielschowsky’s silver stain, × 400)

In addition, examining modified Bielschowsky’s Silver stained sections of the control groups (Ia, Ib, Ic) revealed different degrees of distribution of oligodendroglia cells along the terminal nerve axons with different degrees of myelination. The oligodendroglia appeared as deeply stained circular dots proximal to the nerve axons. Moreover, the myelin sheath appeared as irregular faint blue line surrounding the terminal nerve axons (Fig. 3a, b, c). Tramadol treated groups (IIa, IIb, IIc) exhibited extensive decrease in number of oligodendroglia and extensive demyelination of the terminal nerve axons (Fig. 3d, e, f).

**Fig. 4:**
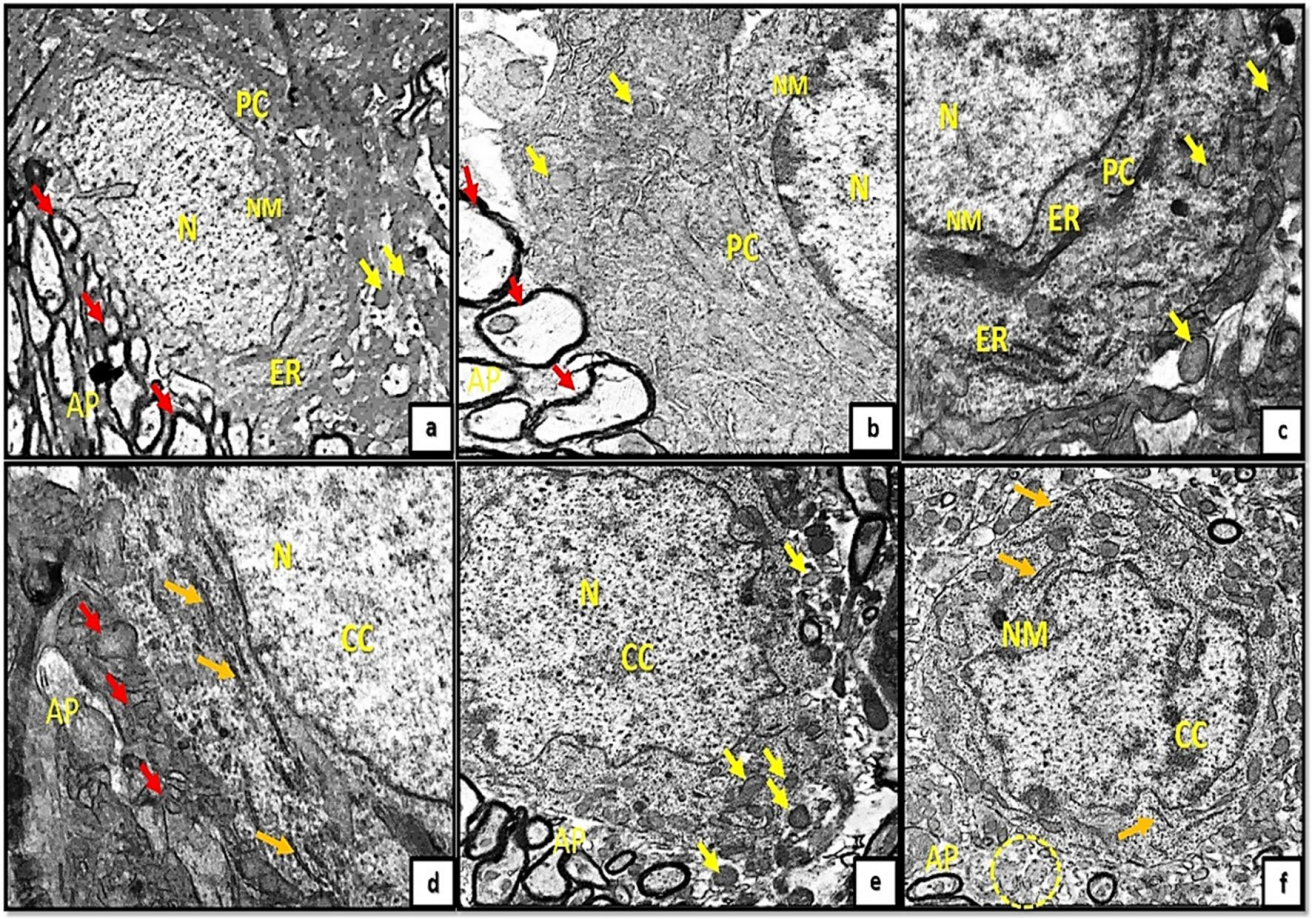
A,4b): photomicrographs of transverse sections of albino rat cerebellar cortex from the control groups (Ia, Ib) showing the purkinje cell(PC) with pale stained nucleus (N) and well defined nuclear membrane (NM),their dendrites surrounded by deeply stained myelin sheath (red arrows) and surrounded by astrocytic processes(AP). Figures (4a,b,C); the cytoplasm of purkinje cell (PC) showing thin filaments of the endoplasmic reticulum(ER), circular mitochondria(yellow arrows).Note also the pale stained nucleus (N) and irregular nuclear membrane (NM).Figs.(4d,4e,4f): transverse section of rat cerebellar cortex from tramadol treated groups (IIa, IIb, IIC) showing purkinje cell with pale stained nuclei(N), clumped chromatin(CC).The cytoplasm showing multiple distorted mitochondria(yellow arrows) and distorted filaments of endoplasmic reticulum(orange arrows).The dendrites appears with pale stainning (red arrows/yellow encirceled area) and surrounded by swollen astrocytic processes (AP) (uranyl acetate& lead citrate × 3000,5000, 7500,5000,5000,5000)

On other hand, examination of cerebellar cortical sections of control groups (Ia, Ib, Ic) by transmission electron microscopy revealed the PCs with pale stained nucleus and well defined nuclear membrane. purkinje cell dendrites appeared surrounded by deeply stained myelin sheath and surrounded by astrocytic processes. Moreover, the cytoplasm showed the filaments of the endoplasmic reticulum, circular mitochondria with well-defined crista (Fig. 4a, b, c). In contrast to tramadol treated groups (IIa, IIb, IIc); PCs appeared with pale stained nuclei, clumped chromatin. The cytoplasm showed multiple distorted mitochondria and distorted filaments of endoplasmic reticulum. The dendrites appeared with pale stainning and surrounded by swollen astrocytic processes (Fig. 4d, e, f).

**Fig. 5:**
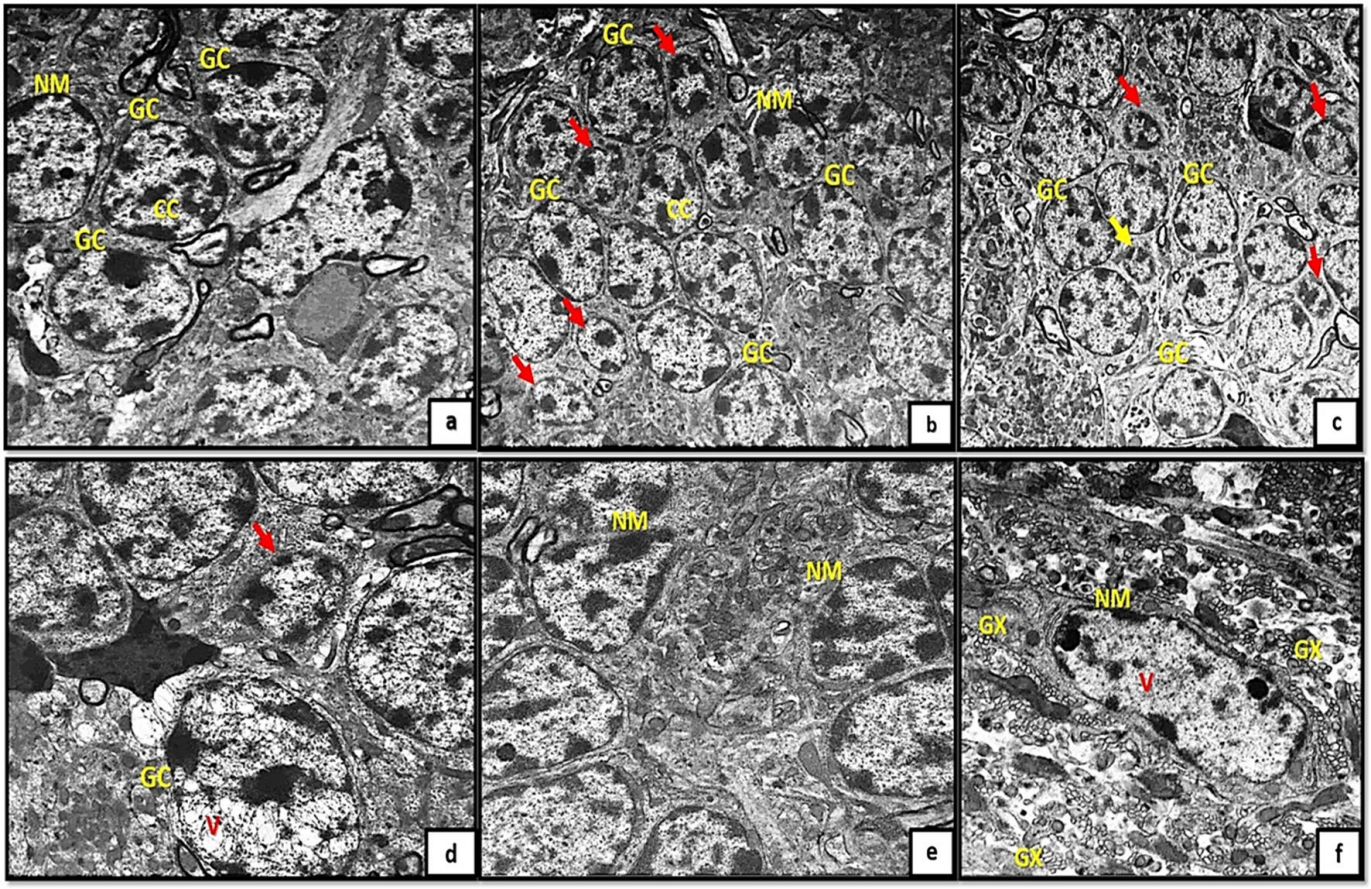
(5a,5b,5c): photomicrographs of transverse section of albino rat cerebellar cortex from the control groups (Ia, Ib, IC) showing granule cells (GC) appear with well-defined circular nuclear membrane(NM) and clumped chromatin (CC). The granule cell precursors have the same shape but appears smaller in size (red arrows). Figures (5d,5e,5f) showing transverse sections of rat cerebellar cortex from tramadol treated groups (IIa, IIb, IIC); the granule cells(GC) appear with extensive vacuolations (V), irregular nuclear membrane(NM), dilated Golgi complex (GX). Note, the marked decrease in number of granule cell precursors (red arrows). (uranyl acetate& lead citrate × 1000, 1000,1000,1500,1500,1500)

Regarding the granule cell layer (GCL); the control groups appeared with well- defined circular nuclear membrane and clumped chromatin. The granule cell precursors have the same shape but appeared much smaller in size (Fig. 5a, b, c). However, in tramadol treated groups the granule cells (GCs) appeared with extensive vacuolations, irregular nuclear membrane, dilated Golgi complex with marked decrease in granule cell precursors (Fig. 5d, e, f).

**Fig. 6:**
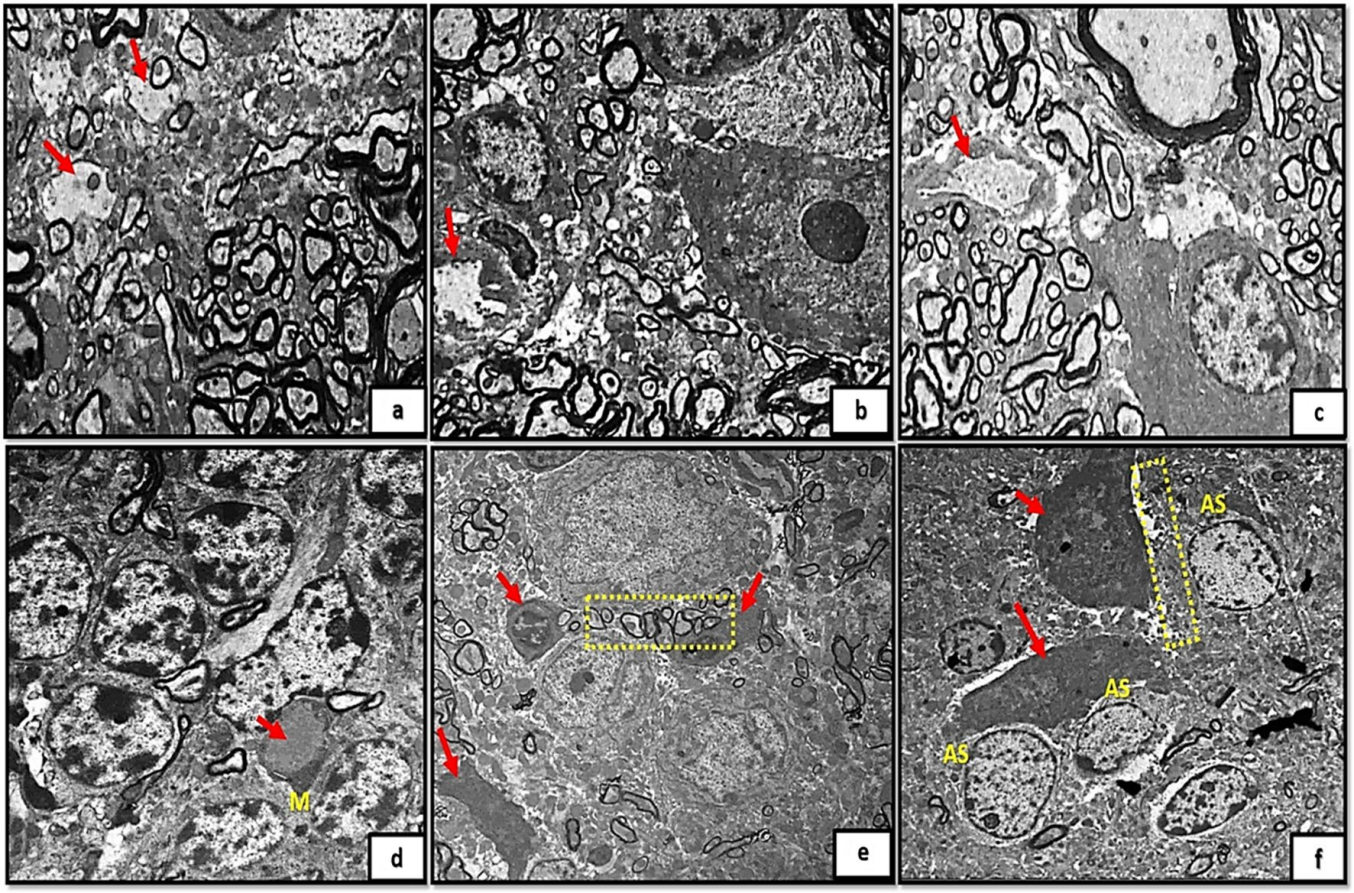
(6a,6b,6c): photomicrographs of transverse sections of albino rat cerebellar cortex from the control groups (Ia, Ib, IC) showing small blood capillaries with thin irregular wall and thin interrupted endothelial linning (red arrows). Figure (6d) showing rat cerebellar cortex from tramadol treated groups (IIa); showing hypertrophic perivascular (red arrow) microglia with deeply stained cytoplasm (M).Figs.(6e, 6f) showing swollen capillaries with obliterated lumen (red arrows).The capillaries mainly surrounded by astrocytes (AS) and astrocytic nerve processes (inset) protruding inside the capillary lumen (uranyl acetate& lead citrate × 3000,1000,3000,1500,1000,1000)

On other hand, the newly formed blood capillaries appeared as small blood capillaries with thin irregular wall and thin interrupted endothelial linning in the control groups (Ia, Ib, Ic) Fig. 6a, b, c. The newly formed capillaries in tramadol treated group (IIa) showed hypertrophic perivascular microglia with deeply stained cytoplasm. While groups IIb, IIc showed swollen capillaries with obliterated vascular lumen. The capillaries appeared mainly surrounded by astrocytes and astrocytic nerve processes protruding inside the capillary lumen (Fig. 6d, e, f).

**Fig. 7:**
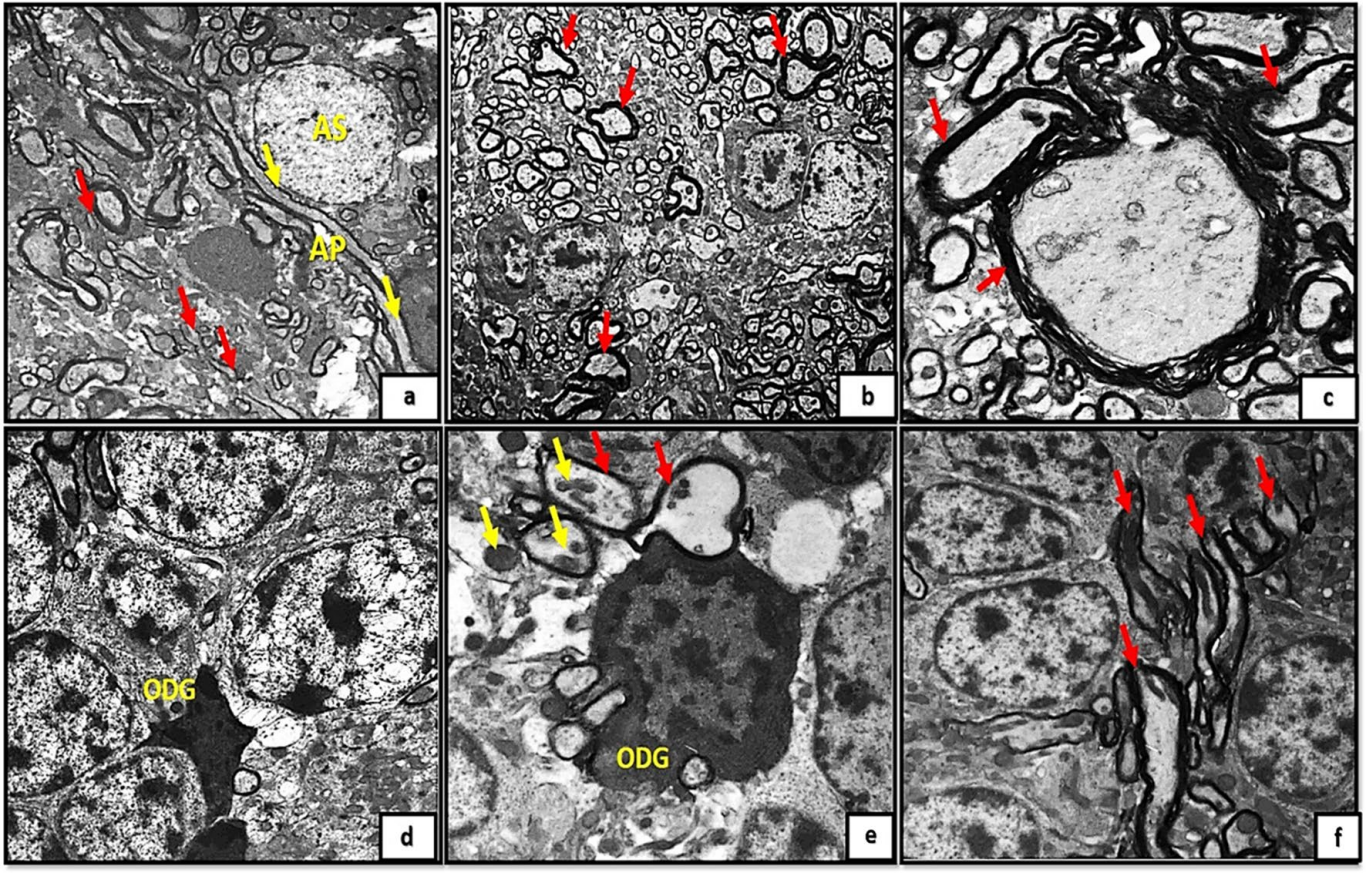
A): A photomicrograph of transverse section of albino rat cerebellar cortex from the control group (Ia) showing the molecular cell layer with multiple newly formed nerve axons with very thin interupted surrounding myelin sheath (red arrows) the astrocyte (AS) appears surrounded also by thin myelin sheaths(AP) (yellow arrows).Figs.(7b,c) showing the molecular cell layer of cerebellar cortex from control groups (Ib, IC);exhibiting terminal axons with thickened laminated myelin sheath (red arrows). Figures (7d,7e,7f) showing rat cerebellar cortex from tramadol treated groups (IIa, IIb, IIC); showing degenerated oligodendroglia (ODG) along with axonal and mitochondrial swellings (yellow arrows) and disrupted myelin figures (red arrows). (uranyl acetate& lead citrate × 3000,1000,3000,1500,2000,1500)

Furthermore, examining sections of group (Ia) showed the MCL with multiple newly formed nerve axons with very thin interupted surrounding myelin sheath (Fig. 7a). The astrocytes appeared surrounded by thin myelin sheath. Groups (Ib, Ic) exhibited the MCL with terminal axons and thickened laminated myelin sheath (Fig. 7b, c). Degenerated oligodendroglia along with axonal and mitochondrial swellings and disrupted myelin figures were clearly observed in tramadol treated groups (IIa, IIb, IIc); Fig. 7d, e, f.

## Immunohistochemical results

**Fig. 8:**
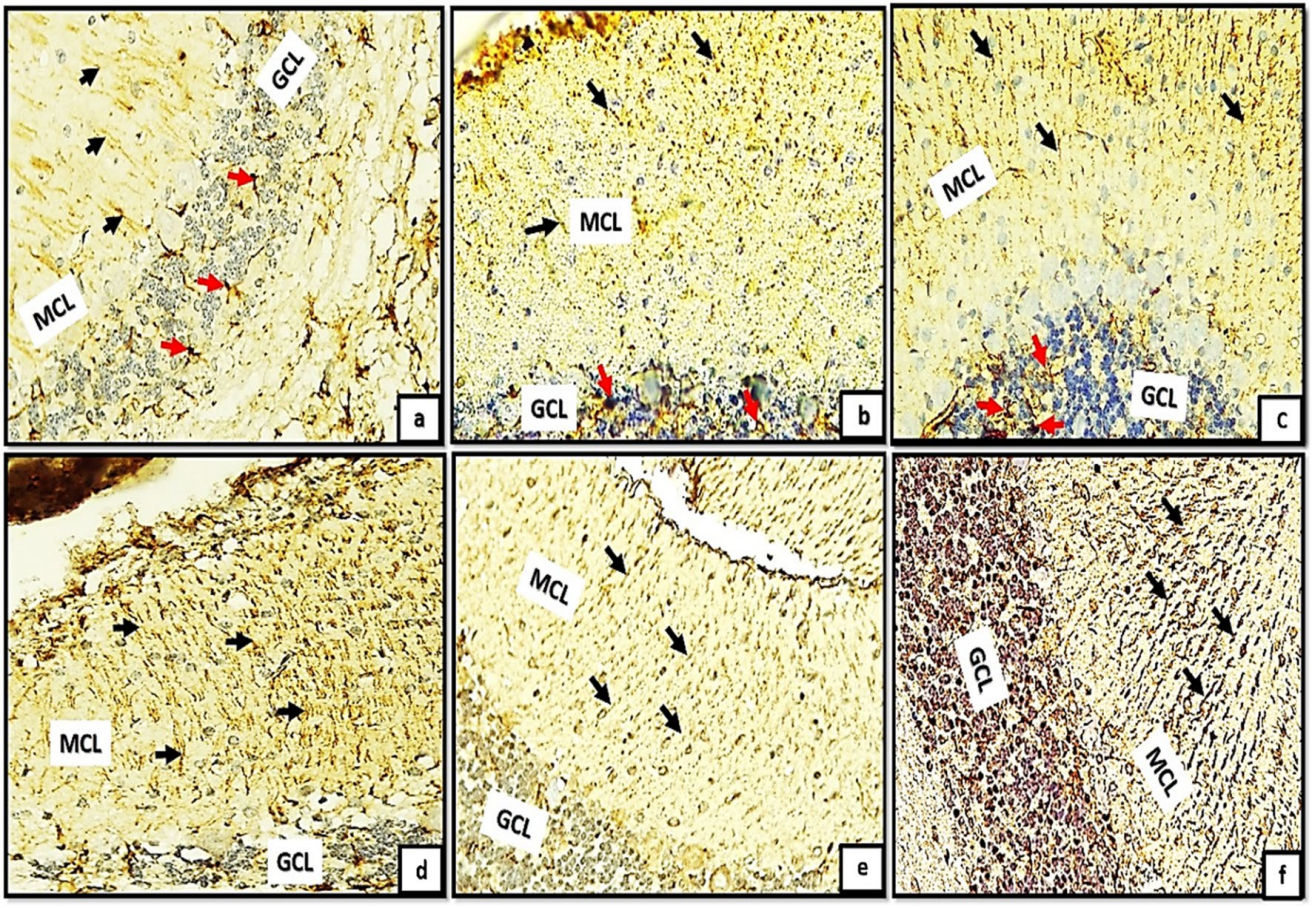

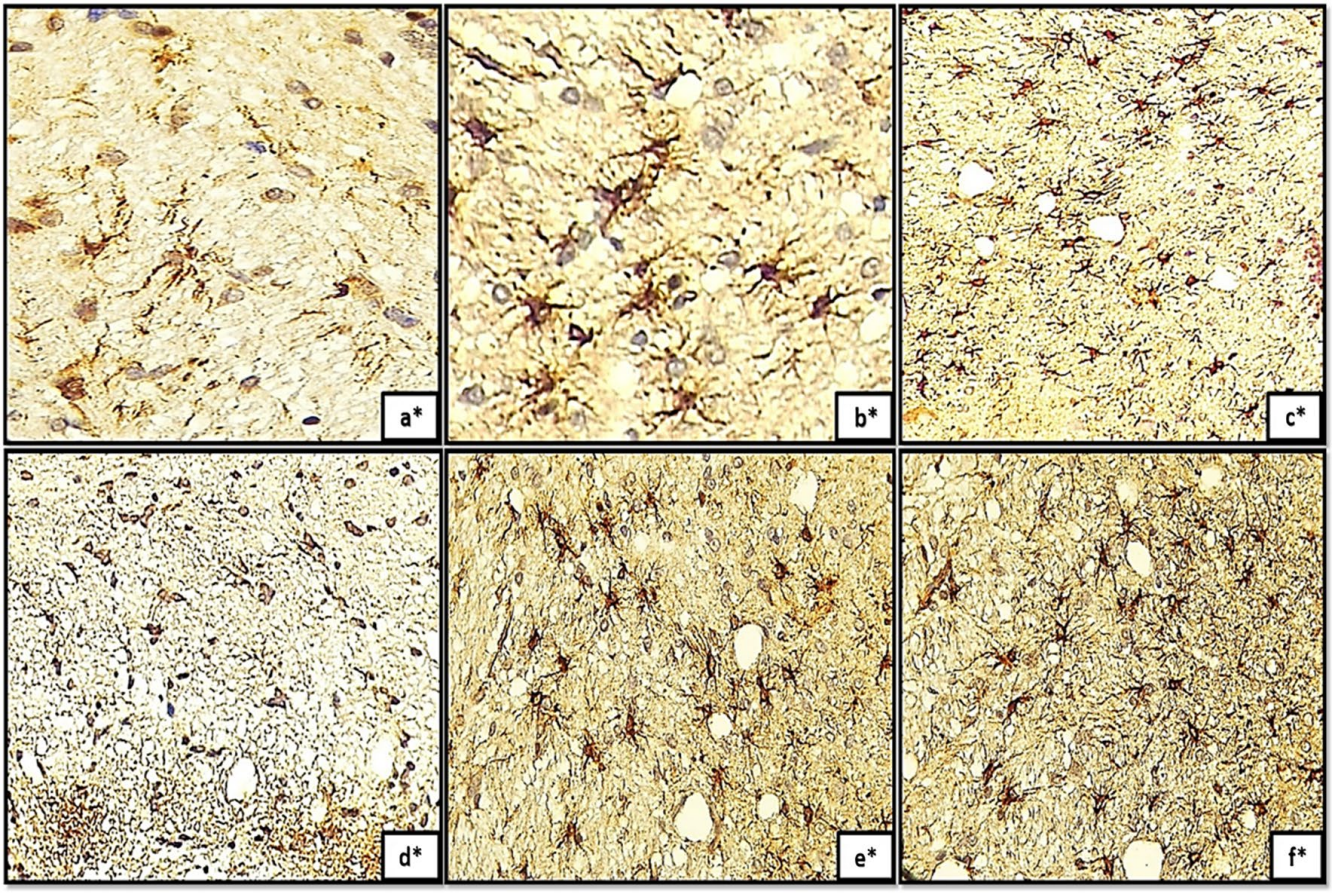
(a,b,c): photomicrographs of transverse section of albino rat cerebellar cortex from the control groups (Ia, Ib, Ic) showing resting astrocytes with their protoplasmic processes in the granular cell layer(red arrows). The astrocytes appear with short processes and moderately-stained cell bodies. In the molecular layer, Bergmann glial fibers were moderately-stained and presented thin, intact morphology (black arrows). Figures (8d,e,f) showing transverse sections of rat cerebellar cortex from tramadol treated groups (IIa, IIb, IIC); expressing strong positive immune reaction to GFAP. The Bergmann glial fibers appeared hypertrophic and intensely-stained (black arrows) in the molecular cell layer (MCL). Note also the difference between the normal expression of astrocytes in the white matter core between control groups Fig. 8a*,b*,c* and the hypertrophic astrogliosis appears in different tramadol treated groups Figs.8d*,8e*,8f* astrocytes in the white matter core(WM) exhibited large cell bodies.(GFAPX400)

In relation to the GFAP-immunoreactivity, the control groups exhibited Bergmann glial fibers with moderate degree of staining and possessed a slender, undamaged appearance within the molecular layer (Fig. 8a, b, c). The groups (IIa, IIb, IIc) demonstrated an exceptional rise in GFAP immunoreactivity, as evidenced by the hypertrophic appearance and intensive staining of Bergmann glial fibers in the MCL (Fig. 8d, e, f). Furthermore, substantial cell bodies were observed not only in the granular layer but also in the white matter nucleus (Fig. 8d*, e*, and f*).

**Fig. 9:**
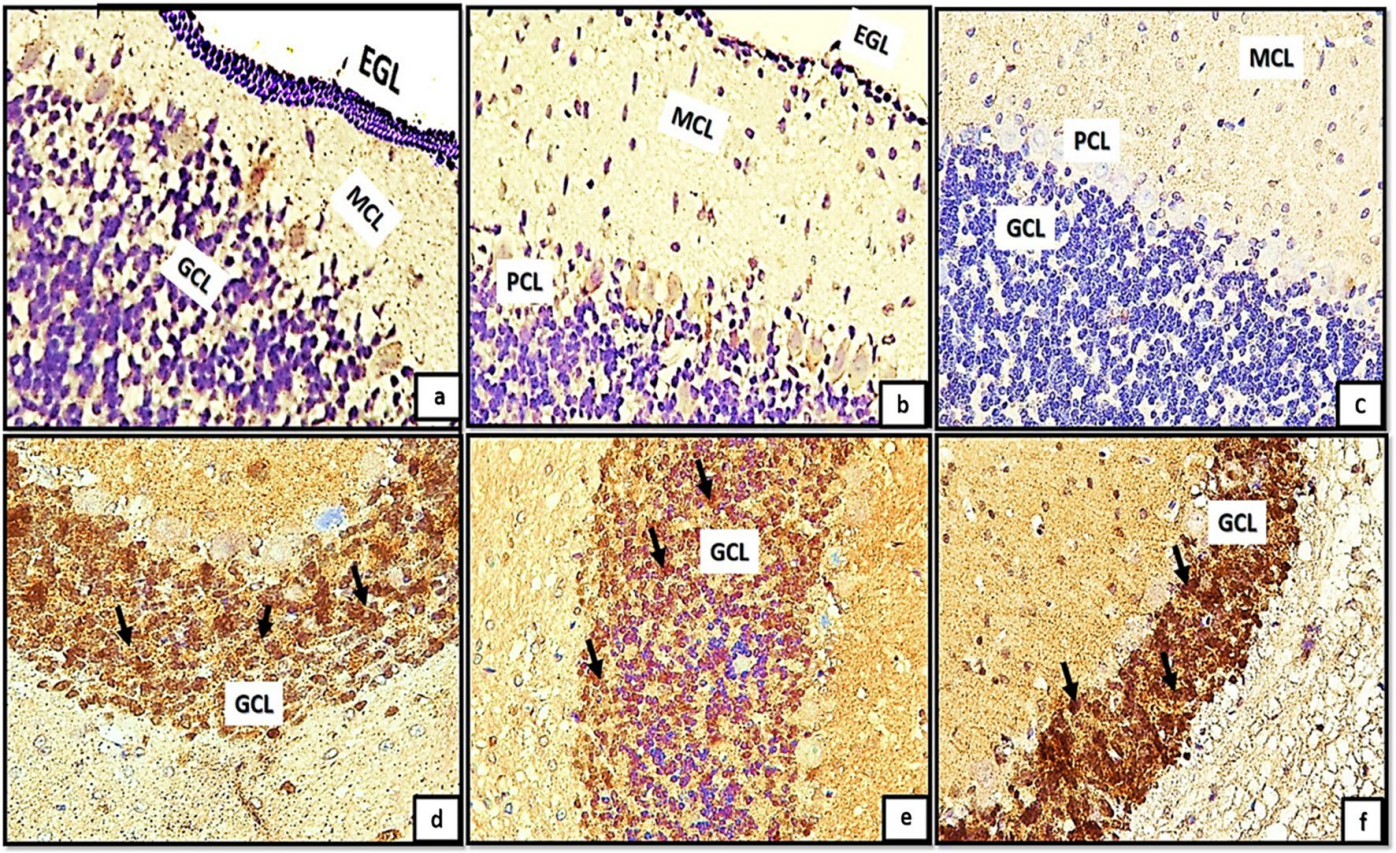
(a,b,c): photomicrographs of transverse sections of albino rat cerebellar cortex from the control groups (Ia, Ib, Ic) showing negative immune reaction; no apoptotic cells could be detected among the three cortical cerebellar layers. Figures (9d,e,f) showing rat cerebellar cortex from tramadol treated groups (IIa, IIb, IIc);multiple brownish and deeply stained apoptotic cells (black arrows) are detected among the granule cell layer(GCL).The reaction appears extensive in groups (IIC)(IId)(Figs.9d,f)(TUNEL assayx400)

Moreover, examining sections of TUNEL assay immunoreactivity of the control groups revealed, negative immune reaction in all cerebellar cortical layers (Fig. 9a, b, c). Despite tramadol treated groups (IIa, IIb, IIc) showed multiple brownish and deeply stained apoptotic cells were detected among the GCL (Fig. 9d, e, f). The reaction appeared extensive in group (IIC, IId) (Fig. 9e, f).

**Fig. 10:**
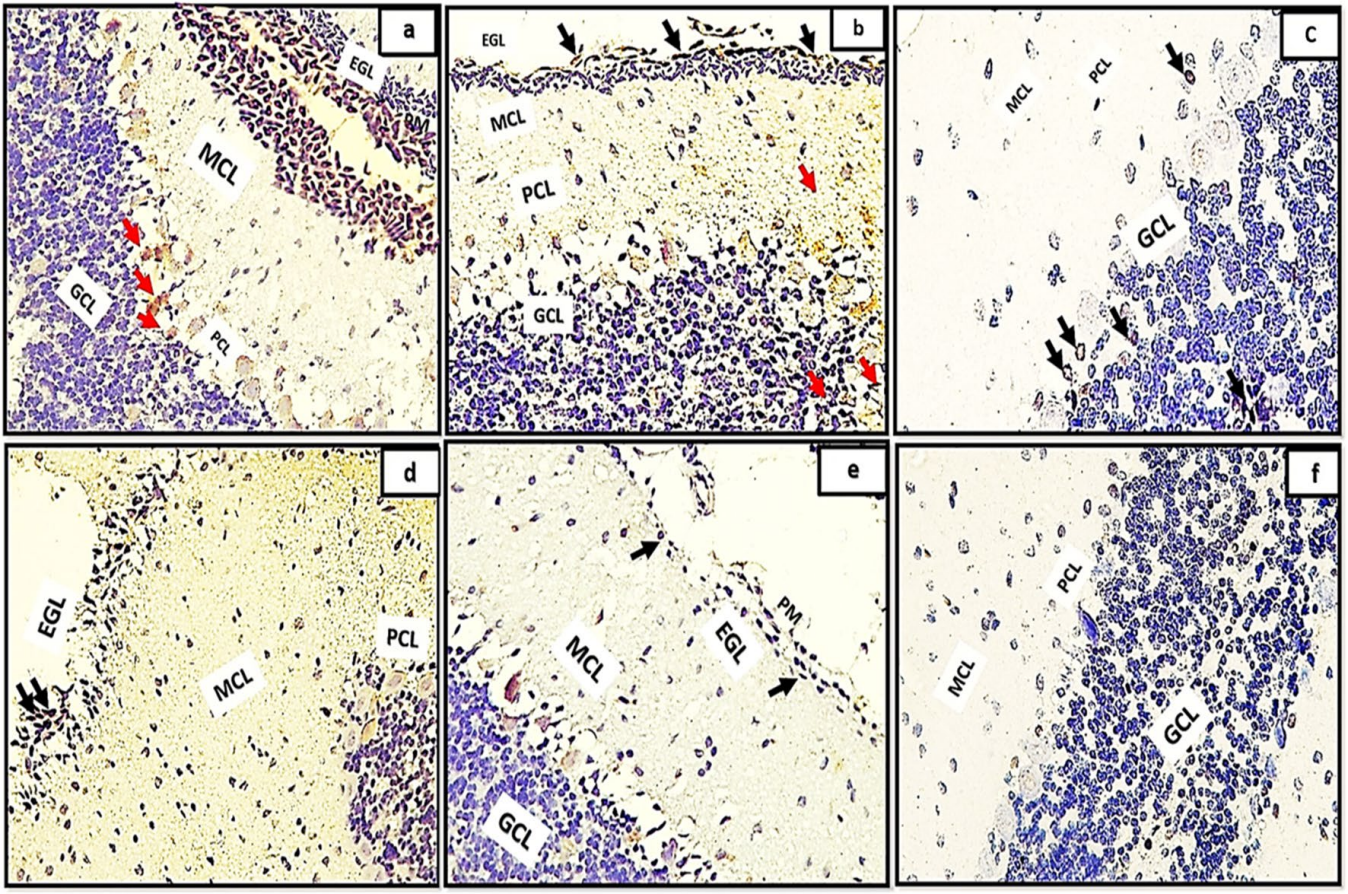
(a,b);groups (Ia,Ib): showing positive immune reaction to Ki67; the reaction appears as brownish cytoplasmic staining of proliferating external granular cells (EGL) black arrows, Bergmann astrocytes in proximity to the purkinje cells (red arrows). Fig. (10c);group (IC): showing positively immune stained cells could be detected in proliferating Bergmann astrocytes and granule cell layer (GCL)(black arrows). Note, progressive decreased reaction among different control groups. Figure (10d); group (IIA): small number of immune positive cells could be detected in external granular layer (EGL). Figures (10e,f); groups (IIB,IIC): showing negative immune reaction to Ki67,no positive immune stained cells are detected through all cerebellar cortical layers.(Ki67 × 400)

Positively immune reacting cells to Ki67; appeared as deeply stained brownish GCs. The reacting proliferating cells were clearly detected mainly throughout the EGL and proliferating Bergmann astrocytes in group (Ia,Ib); Fig. 10a, b. Figure 10c; group (IC): showed positively immune stained cells could be detected in proliferating Bergmann astrocytes and granule cell layer (GCL). Marked decline in the immune positive reaction was clearly detected among different control groups. Besides, marked increase in the thickness of MCL and density of GCL could also clearly shown. Figure 10d; group (IIa): small number of immune positive cells were detected through the EGL. However, groups (IIb, IIc) showed negative immune reaction to Ki67, no positive immune staining cells were detected through all cerebellar cortical layers (Fig. 10e, f.

**Fig. 11:**
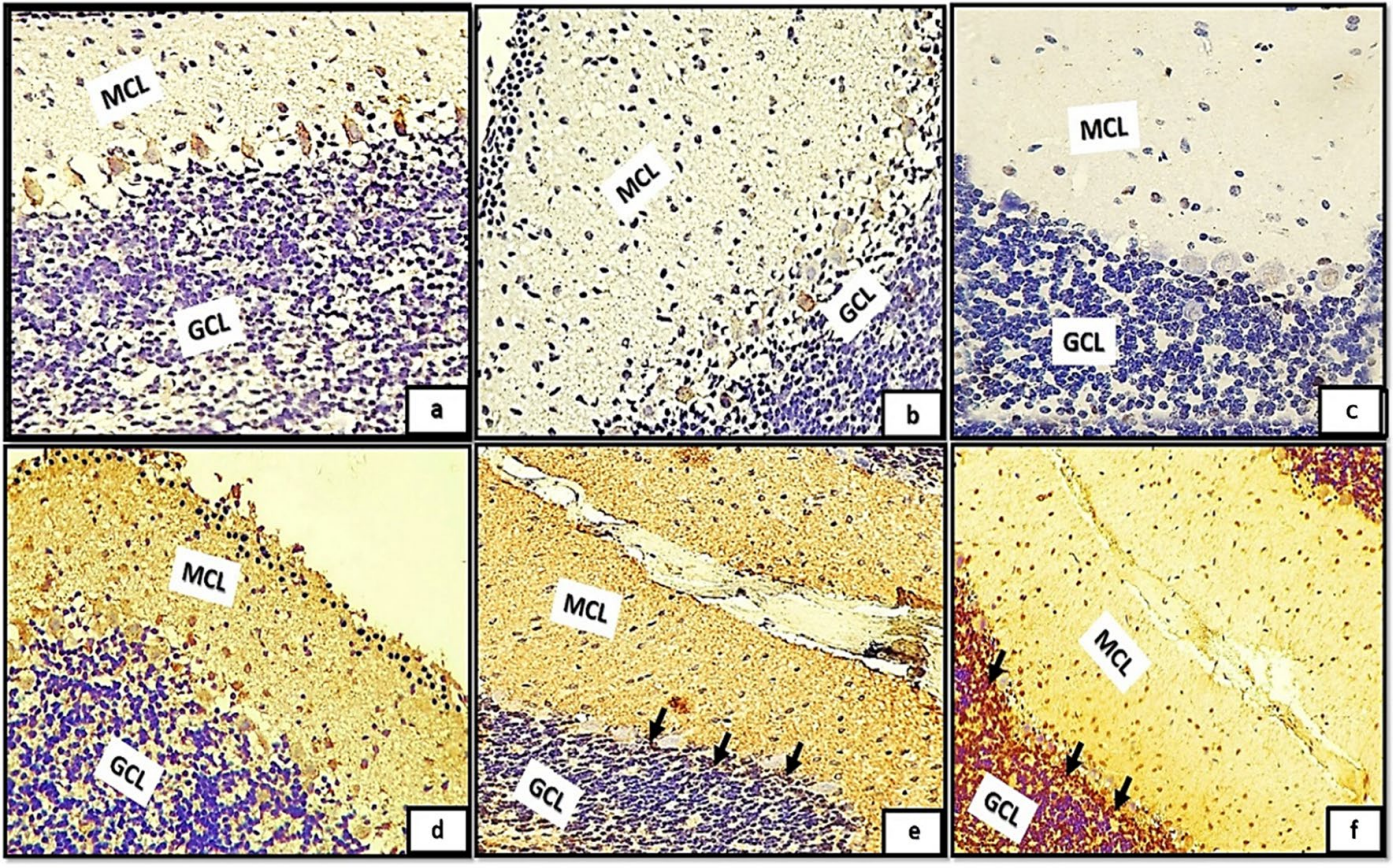
(a,b,C): photomicrographs of transverse sections of albino rat cerebellar cortex from the control groups (Ia, Ib, IC) showing negative p53 immune staining in all cerebellar cortical layers; molecular cell layer (MCL) and granule cell layer (GCL). Figures (11d,e,f) showing transverse sections of rat cerebellar cortex from tramadol treated groups (IIa, IIb, IIC); an increase in P53 immune expression. Figures 11d group IIa; showing positive immune reaction to P53 in the cytoplasm of molecular cell layer (MCL). Figure (11e) showing intense positive immune reaction to P53 in the cytoplasm of both molecular cell layer (MCL) and some superficial cells of granule cell layer (GCL) the (black arrows). Strong positive cytoplasmic immune reaction appears in all layers of cerebellar cortex (Fig. 11f) (P53 × 400)

On other hand, examining sections of P53 immunoreactivity exhibited negative immune staining in all cerebellar cortical layers (Fig. 11a, b, c). While tramadol treated groups (IIa, IIb, IIc); showed an extensive increase in P53 immune expression. Figure 11d group IIa; showed positive immune reaction to p53 in the cytoplasm of molecular cell layer. Figure 11e showing intense positive immune reaction to p53 in the cytoplasm of both MCL and GCL. Strong positive cytoplasmic immune reaction appears in all cerebellar cortical layers (Fig. 11f).

### Biochemical results

#### Statistical results of MDA levels in rat serum PND 21 (Table 1/Histogram 1)

**Table 1:** SOD and MDA levels concentration (Pg/ml) in serum of rats from control group Ic and tramadol group IIc (One-way ANOVA, p < 0,05)

		Control group	Tramadol group	Test value	*P*-value	Sig
		No. = 5/each subgroup	No. = 5/each subgroup			
SOD level conc. (Pg/ml)	Mean ± SD	110.66 ± 6.14	18.02 ± 2.18	37.644•	0.000	HS
	Range	104.55 – 120.33	14.33 – 20.77			
MDA level conc. (Pg/ml)	Mean ± SD	33.73 ± 4.41	563.24 ± 79.97	-17.491•	0.000	HS
	Range	28.62 – 39.4	487.33 – 700.44			

**Histogram 1:**
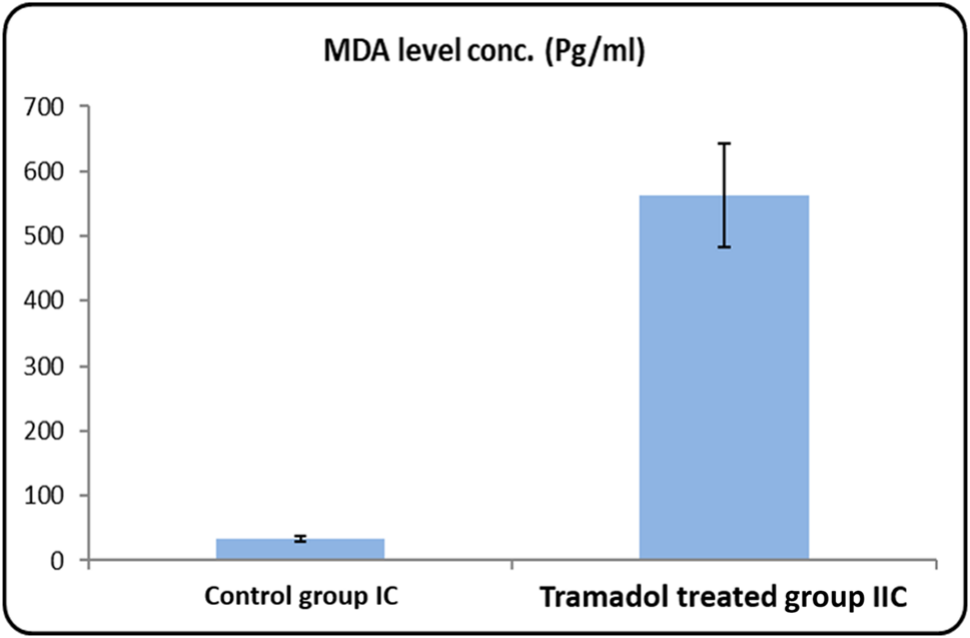
MDA levels concentration (Pg/ml) in serum of rats from control group Ic and tramadol group IIc

Estimated serum MDA levels clarified that there was a significant increase in the serum MDA level in PND21 tramadol treated group in comparison to its matched control group (P-value < 0.05).

#### Statistical results of SOD levels in rat serum PND21 (Table 1/Histogram 2)

**Histogram 2:**
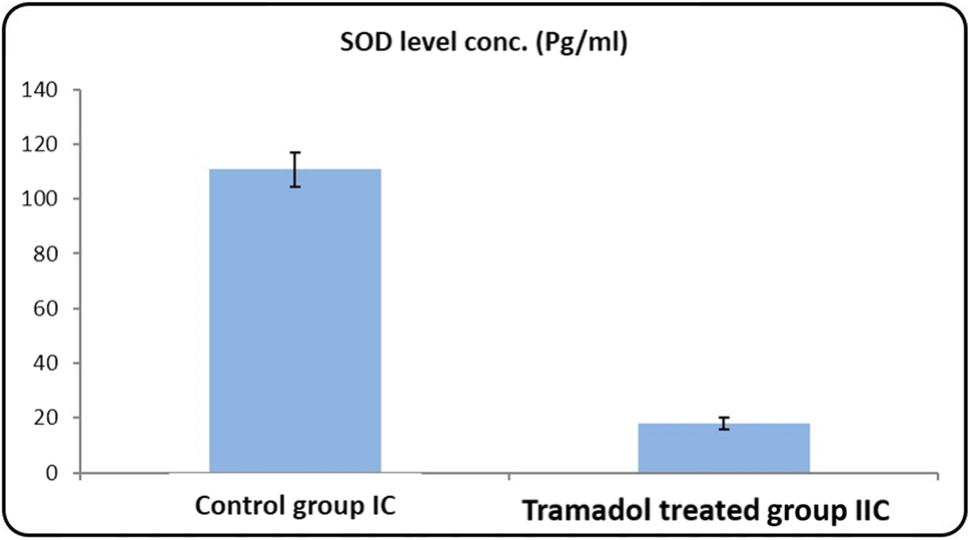
SOD levels concentration (Pg/ml) in serum of rats from control group Ic and tramadol group IIc

In contrast, estimated serum SOD levels clarified that there was a significant decrease in the serum SOD level in PND21 tramadol treated group in comparison to its matched control group (*P*-value < 0.05).

### Morphometric results

#### Measurement of thickness of the external granular cell layer (EGL) in micrometer (µm) (Table 2/ Histogram 3)

**Table 2:** Comparison between control and tramadol groups regarding the estimated thickness of external granular cell layer (µm) (One-way ANOVA, *p* < 0,05)

Estimated thickness in (µm)		Control group	Tramadol group	Test value	*P*-value	Sig
		No. = 5/ each group	No. = 5/ each grou**p**			
Day 7	Mean ± SD	28.44 ± 0.77	27.07 ± 0.76	-1.536•	0.150	NS
	Range	26.33 – 28.6	26.77 – 29.01			
Day 14	Mean ± SD	25.12 ± 0.97	29.41 ± 2.22	-4.684•	0.001	HS
	Range	23.45 – 26.51	26.41 – 32.44			
Day 21	Mean ± SD	18.39 ± 1.18	29.71 ± 5.15	-5.675•	0.000	HS
	Range	16.85 – 19.99	19.22 – 33.33			
**Repeated Measures ANOVA test**	**F**	297.548	0.893			
	***P-value***	< 0.001	0.387			

**Histogram 3:**
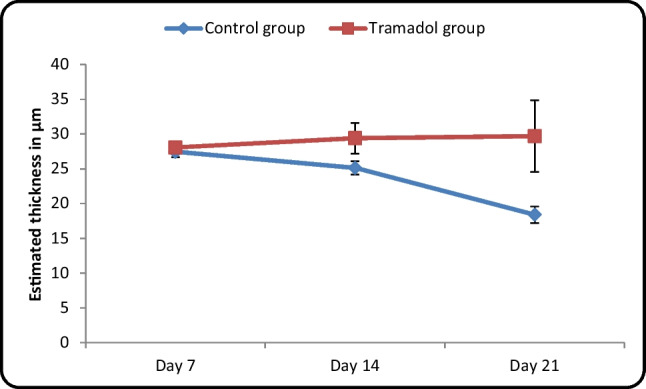
Comparison between control and tramadol groups regarding the estimated thickness of external granular cell layer (µm)

Estimation of thickness of EGL in the control groups revealed that there was a significant decrease between different control groups PND7, PND14, PND21 with (*P*-value < 0.05) and no significant decrease between different tramadol treated groups with (*P*-value > 0.05). On other hand, there was a significant decrease in thickness of EGL of tramadol treated groups PND14, PND21 in comparison to their matched controls (*P*-value < 0.05).

#### Measurement of the thickness of the Molecular cell layer (MCL) in micrometer (µm) (Table 3/Histogram 4):

**Table 3:** Comparison between control and tramadol treated groups regarding the thickness of molecular cell layer (µm) (One-way ANOVA, *p* < 0,05)

Estimated thickness in (µm)		Control group	Tramadol group	Test value	*P*-value	Sig
		No. = 5/each subgroup	No. = 5/each subgroup			
Day 7	Mean ± SD	42.58 ± 2.55	30.09 ± 1.83	10.518•	0.000	HS
	Range	40.3 – 46.44	27.66 – 33.22			
Day 14	Mean ± SD	105.63 ± 2.55	89.28 ± 4.51	8.355•	0.000	HS
	Range	102.44 – 109.9	83.11 – 95.44			
Day 21	Mean ± SD	137.06 ± 1.44	111.93 ± 4.47	14.152•	0.000	HS
	Range	134.98 – 139.22	106.55 – 117.99			
**Repeated Measures ANOVA test**	**F**	2545.261	666.245			
	**P-value**	0.000	0.000			

**Histogram 4:**
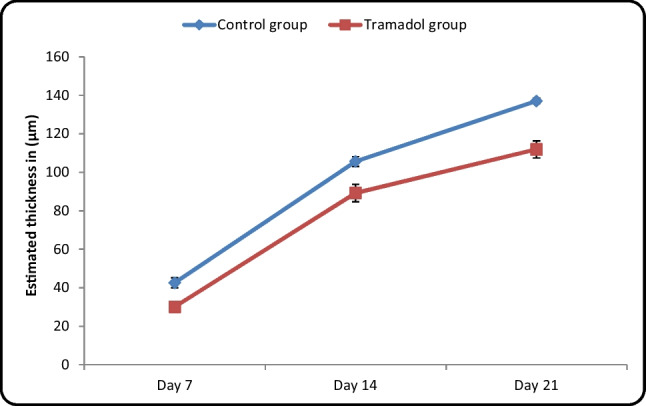
Comparison between control and tramadol treated groups regarding the thickness of molecular cell layer (µm)

Estimation of thickness of MCL revealed a significant increase between different control groups with (*P*-value < 0.05). Moreover, there was a significant increase between different tramadol treated groups (P-value < 0.05). The estimated thickness of MCL in control groups PND7, PND14, PND21 showed a significant increase in comparison to their age matched tramadol treated groups PND7, PND14, PND21 with (*P*-value < 0.05).

#### Measurement of the thickness of the Internal granular cell layer (IGL) in micrometer (µm) (Table 4/Histogram 5)

**Table 4:** Contrast between control and tramadol groups regarding the estimated thickness of internal granular cell layer (µm) (One-way ANOVA, p < 0,05)

Estimated thickness in (µm)		Control group	Tramadol group	Test value	P-value	Sig
		No. = 5/each subgroup	No. = 5/each subgroup			
Day 7	Mean ± SD	97.32 ± 1.96	94.97 ± 2.37	14.348•	0.000	HS
	Range	94.33 – 99.55	92.11 – 99.44			
Day 14	Mean ± SD	103.97 ± 2.15	88.90 ± 1.35	7.435•	0.000	HS
	Range	100.44 – 106.5	86.33 – 90.44			
Day 21	Mean ± SD	104.02 ± 3.57	82.98 ± 1.77	10.493•	0.000	HS
	Range	100.44 – 109.22	80.55 – 85.66			
**Repeated Measures ANOVA test**	**F**	10.241	56.776			
	**P-value**	0.018	0.000			

**Histogram 5:**
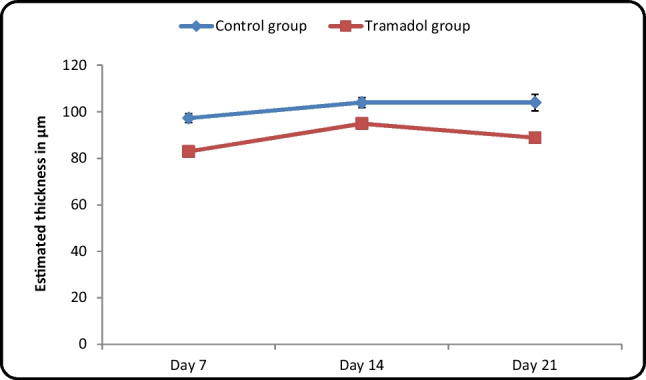
Comparison between control and tramadol groups regarding the thickness of internal granular cell layer (µm)

Estimated thickness of IGL revealed no significant increase between different control age groups (*P*-value < 0.05). Moreover, there was a significant decrease in the estimated thickness of IGL between different tramadol treated groups with (*P*-value < 0.05). In addition, the estimated thickness of (IGL) showed a high significant decrease in tramadol treated groups PND7, PND14, PND21 in comparison to their age matched treated groups PND7, PND14, PND21 with (*P*-value < 0.05).

#### Mean area % of apoptotic cells in different cerebellar cortical layers estimated by TUNEL assay (Table 5/Histogram 6):

**Table 5:** Comparison between control and tramadol groups regarding the area% of immune stain (TUNEl immunohistochemistry) (One-way ANOVA, *p* < 0,05)

TUNEl immunohistochemistry %		Control group	Tramadol group	Test value	*P*-value	Sig
		No. = 5/each subgroup	No. = 5/each subgroup			
Day 7	Mean ± SD	1.69 ± 0.22	2.28 ± 0.46	-3.067•	0.010	S
	Range	1.46 – 2.05	1.59 – 2.88			
Day 14	Mean ± SD	1.71 ± 0.21	2.84 ± 0.53	-5.178•	0.000	HS
	Range	1.5 – 2.07	1.99 – 3.55			
Day 21	Mean ± SD	1.69 ± 0.23	2.94 ± 0.57	-5.394•	0.000	HS
	Range	1.43 – 2.07	1.953.59			
**Repeated Measures ANOVA test**	**F**	6.351	6.033			
	**P-value**	0.016	0.043			

**Histogram 6:**
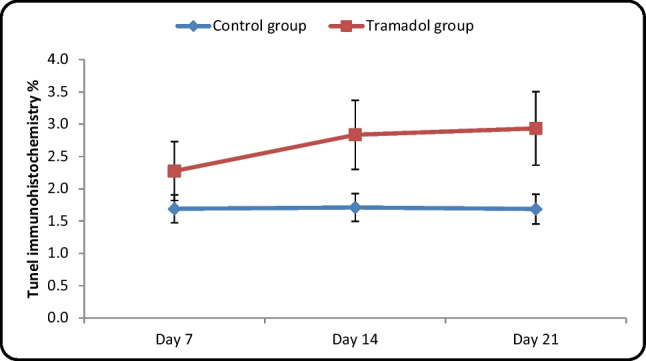
Comparison between control and tramadol groups regarding the area% of immune stain (TUNEl immunohistochemistry)

Regarding the area percentage of apoptotic cell distribution in different cortical cerebellar layers; there wasn't a significant difference in the mean area percentage of apoptotic cell distribution between different control groups (Ia, Ib. Ic) (*P*-value > 0.05). Moreover, there was a significant difference between different tramadol treated groups (IIa, IIb, IIc) (*P*-value < 0.05). In contrast, there was a significant increase in the mean area percentage of apoptotic cell distribution in tramadol treated group IIa in comparison to its matched control age group. Finally, a significant increase in the mean area percentage of apoptotic cell distribution was also detected between groups (IIb, IIc) and their matched control groups (*P*-value < 0.05).

#### Mean area % of GFAP in different cerebellar cortical layers (Table 6/Histogram 7)

**Table 6:** Contrast between control and tramadol groups regarding the area % GFAP immunohistochemistry (One-way ANOVA, *p* < 0,05)

GFAP immunohistochemistry %		Control group	Tramadol group	Test value	*P*-value	Sig
		No. = 5/each subgroup	No. = 5/each group			
Day 7	Mean ± SD	0.37 ± 0.16	4.98 ± 1.30	-9.279•	0.000	HS
	Range	0.15 – 0.63	2.94 – 6.65			
Day 14	Mean ± SD	0.40 ± 0.16	4.98 ± 0.73	-16.270•	0.000	HS
	Range	0.18 – 0.66	3.92 – 5.77			
Day 21	Mean ± SD	0.39 ± 0.17	5.19 ± 0.79	-15.687•	0.000	HS
	Range	0.16 – 0.68	3.95 – 6.01			
**Repeated Measures ANOVA test**	**F**	12.422	0.339			
	**P-value**	0.004	0.588			

**Histogram 7:**
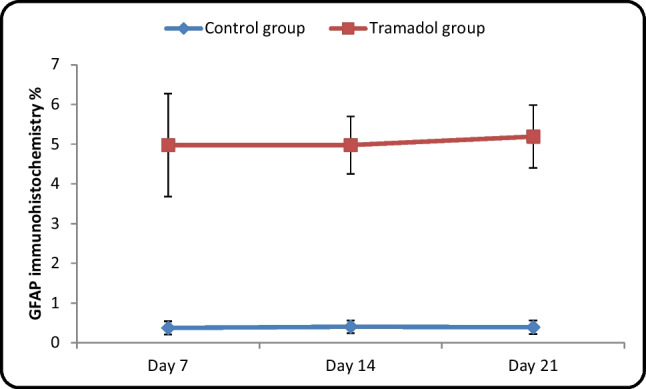
Contrast between control and tramadol groups regarding the area % GFAP immunohistochemistry

Regarding GFAP immuno reactivity, there was a significant difference in the mean area percentage of GFAP immune expression in different cerebellar cortical layers between different control groups (Ia, Ib, Ic) (*P*-value < 0.05). Moreover, there wasn't a significant difference between different tramadol treated groups (IIa, IIb, IIc). On other hand, there was a significant increase in area percentage of GFAP immune expression between tramadol treated groups and their matched controls (*P*-value < 0.05).

#### Quantitative PCR gene expression of Micro-RNA7 in different cortical cerebellar layers (Table 7/Histogram 8)

**Table 7:** Comparison between control and tramadol groups regarding Micro-RNA7 gene expression in cortical cerebellar layer (One-way ANOVA, p < 0,05)

Micro- RNA7 gene expression in cortical cerebellar layer		Control group	Tramadol group	Test value	P-value	Sig
		No. = 5/each subgroup	No. = 5/each subgroup			
Day 7	Mean ± SD	30.01 ± 2.23	27.93 ± 1.54	2.028•	0.055	S
	Range	27.88 – 34.22	26.22 – 30.12			
Day 14	Mean ± SD	34.21 ± 1.66	27.59 ± 1.68	7.408•	0.000	HS
	Range	32.33 – 36.11	25.33 – 29.62			
Day 21	Mean ± SD	36.01 ± 2.23	27.93 ± 1.54	2.028•	0.000	HS
	Range	27.88 – 34.22	26.22 – 30.12			
**Repeated Measures ANOVA test**	**F**	16.782	2.407			
	**P-value**	**0.006 (HS)**	0.172 (NS)			

**Histogram 8:**
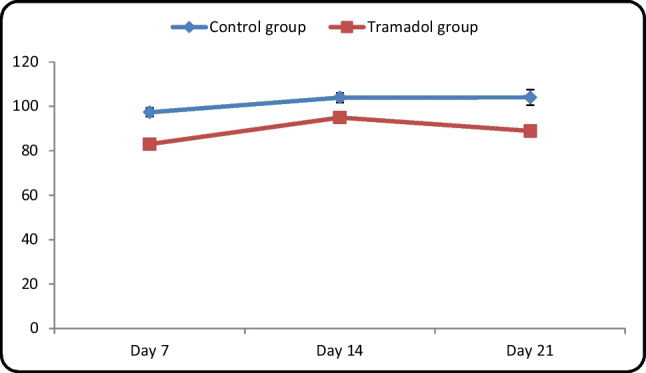
Comparison between control and tramadol groups regarding Micro-RNA7 gene expression in cortical cerebellar layer

Regarding control groups, quantitative PCR results clarified that there was a significant difference between different control groups PND7, PND14, PND21 with (*P*-value < 0.05). In contrast, there wasn't a difference between different tramadol treated groups with (*P*-value > 0.05). Furthermore, there was a statistical significant decrease in Micro-RNA gene expression between tramadol treated PND7 and its matched control group. In addition to the significant decrease in MiR-7 expression in tramadol treated groups PND14, PND21 and their matched control groups (*P*-value < 0.05).

## Discussion

The incidence of prenatal drug exposure is increasing worldwide, exhibiting considerable variation among nations (Falsaperla et al. 2020). Development of the fetus's brain occurs during pregnancy. During the first trimester of pregnancy, critical developmental processes occur (Singer et al. 2020). Early-life drug and substance exposure has enduring detrimental impacts on the structure and function of the brain (Martin et al. 2016). Embryonic development-related exposure to addictive substances and drugs can cause modifications to the cellular structure of neurons in the cortex. Synaptic plasticity, receptor function and neuronal architecture of numerous excitatory and inhibitory neurons in the marginal system of the midbrain cortex are altered by drug use (Li et al. 2020). Experimental and animal studies continue to demonstrate that prenatal neurodevelopmental disturbances continue to impact the CNS development of fetuses, neonates, infants and adolescents (Corsi et al. 2020). Recent research has focused on elucidating the correlation between prenatal substance exposure and the subsequent neurodevelopmental outcomes of children (Lee et al. 2020).

The purpose of the present investigation is to illustrate the cerebellar changes induced by tramadol during the critical period of synaptic development and neuronal differentiation, which is restricted to the initial three weeks after birth. In the present study, H&E stained sections revealed the general architecture of the cerebellar cortex which appeared consisting of three different layers' EGL, MCL and IGL. In PND7; the EGL appeared extensively thick and the GCs appeared migrating from the EGL towards the IGL through the MCL. Depending on that fact; the EGL appeared thick in PND7 and progressively decreased in thickness in both PND14 and PND21. Besides, the IGL showed progressive increase in its thickness in different control groups. Moreover, MCL appeared with progressive increase in its thickness from PND7 to PND21 respectively.

Concerning purkinje neurons, they are considered the cerebellar cortex's cornerstone, around which cerebellar circuits are processed and conducted. In H&E-stained sections from PND7, Purkinje neurons appeared mitotic, densely populated, and arranged in multiple layers, whereas in PN14, PND21, they appeared arranged in a single layer and adopted their characteristic shape and arrangement. There were Bergmann astrocytes in close proximity to purkinje neurons and their protoplasmic processes. It is well known that the GCs migrates from the EGL to the IGL as a consequence of these protoplasmic processes, which extend extensively throughout the MCL. Synaptogenesis, dendritogenesis, and the maturation of Purkinje and granule neurons are further processes that are correlated with Bergmann glial cell cytodifferentiation (Aboulhoda and Hassan 2018).

In tramadol treated groups; the EGL appeared with decreased thickness or even hardly detected in some sections of different stages of development PND7, PND14, PND21. MCL showed progressive decrease in thickness in comparison to its corresponding control group. The reduction in MCL thickness may be attributed to the deterioration and reversal of dendritic arporizations of Purkinje neurons. On other hand, the progressive decrease in thickness of the GCL could be explained by defective neurogenesis affecting the EGL and defective migration of cells from EGL to IGL.

Furthermore, the reduction in IGL thickness may be accounted for by tramadol's inhibitory effect on the cyclin-dependent kinase system; Vriens et al. (2009) established the intimate relationship between this system and cell division, apoptosis, differentiation, and nervous system function; their research revealed that this relationship disrupts the kinetics of the cell cycle in cerebellar granule progenitors***.*** Opioids inhibit the development of precursor neurons of the cerebellar granule layer in rodents and have been observed to promote cell apoptosis and decrease DNA synthesis in rat cerebellum granule layer neuroblasts, according to in vitro studies (Hauser et al. 2000). This results in detrimental consequences for the survival and differentiation of cerebellar granule cells, essentially impeding signaling events (Ezi et al. 2021).

PCL showed extensive degenerative signs and appeared highly crowded especially at PND14 and PND21. Moreover, they appeared with downward displacement in the granular layer and disposed at various levels in groups of PND21. This crowding could be explained by a trial of PCs to re-establish the synaptic contact with other neurons.

In relation to the immunoreactivity of P53, the present investigation unveiled that groups of rats treated with tramadol demonstrated a notable upregulation in the P53 immune-expression. The PND7 group exhibited a favorable immune response towards P53 within the molecular cell layer's cytoplasm. PND14 exhibited a pronounced positive immune response to P53 in both the GCL and MCL of the cytoplasm. Furthermore, in PND21, a burst of positive cytoplasmic immune response was observed in every stratum of the rat cerebellar cortex, including the white matter. Interestingly; the rate of apoptosis detected by TUNEL assay was greatly elevated in tramadol treated groups in comparison to the corresponding control groups. In addition, Ki67 immunoreactivity was estimated for detection of rate of cell proliferation in the cell progenitors. There was significant decrease in the area percentage stained by Ki67 among tramadol treated groups in comparison to their corresponding control groups. The previous results could be attributed to the fact that oxidative stress could induce a DNA damage, cell cycle arrest and decreased Ki-67 labeling (Cerri et al. 2011).

P53 operates as a signaling hub and is a dynamic transcription factor (Kamada et al. 2016)*.* A critical regulator of cell growth, differentiation, deoxyribonucleic acid (DNA) repair, and apoptosis in numerous stressful situations. (Brož and Attardi 2010; Wang and Sheetz 2023). The P53 gene inhibits DNA repair by delaying cell division and halting the cell cycle at the conclusion of the G1 phase in response to DNA damage (Siganaki et al. 2010). P53 levels decline until DNA repair is completed and the cycle is complete. P53 stimulates the transcription of pro-apoptotic genes and induces cellular apoptosis in the event that DNA repair is unsuccessful (Hoda and Hoda 2020).

In addition, consistent gene expression regulation governs the proper development of the cerebral cortex in mammals, which is vital for the proper operation of the brain. Thus, the results of the present study demonstrated the close relationship between microRNA7 gene expression and typical cerebellar cortex development. The quantitative PCR findings indicated a noteworthy reduction in the expression of the micro RNA7 gene in groups treated with tramadol across a range of age groups, when compared to the control groups. This may be closely associated with the elevated levels of P53 immune expression and apoptosis rate detected by the TUNEL assay, as well as the reduced expression of Ki67 in progenitors of active proliferating cells.

The fundamental regulation of cell-cycle arrest, cell death, and neuronal cell differentiation by micro RNA-7 is well established (Prodromidou and Matsas 2022). It was discovered that five predicted target genes of miR-7 are linked to the P53 signaling pathway and regulate neural development survival or differentiation; among these is the cytosolic adenylate kinase Ak1, which is involved in neuronal differentiation. Pmaip1, also referred to as Noxa, is an activator of apoptosis. Cyclone Ccng, the transcription factor Klf4, and Cdkn1a (also known as p21). MiR-7 function inhibition significantly attenuated the expression of each of the antecedent genes, as is well documented in prior research (Zolboot et al. 2021).

In their study, Pollock et al. (2014) developed a mouse model wherein a miR-7 sponge is employed to precisely inhibit the activity of miR-7 in the cerebral cortex. These findings align with those of the present investigation. As evidenced by the results, the cortices of the miR-7 sponge mice are considerably smaller. The findings of this study indicate that the transition of radial glial cells (RGC) to intermediate progenitor cells (IP) and the survival of progenitors in the subventricular zone are dependent on MiR-7 activity.

On the other hand, miR-7 function is carried out at least in part via direct regulation of P53 pathway genes such as Ak1 and p21, which promote cell-cycle arrest and can lead to apoptosis and plays a crucial role in controlling cortical size among noncoding RNAs. MiR-7 modulates the expression levels of multiple targets, thereby playing a crucial role in cortical neurogenesis, according to the findings of the present study. In order to control normal brain size, the regulation of p53 pathway targets Ak1, p21 and possibly others enable proper RGC-to-IP transition, prevents progenitor apoptosis and permits subsequent neuronal production.

Additionally, transmission electron microscopy (TEM) analysis of sections unveiled indications of degeneration. Neurodegenerative attributes were observed in the purkinje neurons, including chromatin condensation, dilated Golgi channels, and conspicuous infolding of the nuclear envelope. The results presented here align with previous studies that documented the neurotoxicity and degeneration of red neurons induced by tramadol. The degenerated, reddish appearance of PCs observed under light microscopy can be sufficiently explained by ultrastructural observations of chromatin condensation. Perikarya of PCs in which tramadol was administered demonstrated an abnormal aggregation of mitochondria with an abnormal morphology. PCs employ a mechanism that is nearly identical to this as a compensatory response to harmful stimuli and free radicals.

The findings of this study are consistent with those of Gholami et al. (2023), who demonstrated that administration of tramadol to the rat hippocampus could induce neuronal degeneration and apoptosis via inhibition of the TNF- or IL-1β/JNK/Bcl-2/Beclin1 and Bcl-2/Bax signaling pathways and dysfunction of mitochondrial respiratory chain enzymes. In addition, the present study revealed that some nerve axon mitochondria were enlarged and degenerated. Tramadol-treated groups exhibited notable mitochondrial alterations, including an abundance of vacuolated and enlarged mitochondria within degenerated neurons. Oxidative stress-induced disruption of mitochondrial cristae is widely recognized to result in a reduction in energy production and the initiation of DNA fragmentation (Nguyen et al. 2023). Similar mitochondrial alterations have been observed in the brains of tramadol-treated rats in earlier studies (Omar 2016) and these alterations were stopped by antioxidant therapy. Although oxidative stress and mitochondrial structural changes have been linked, it is still possible that mitochondrial damage leads to oxidative stress.

Tramadol has been shown in other research to inhibit the complexes of the mitochondrial electron transport chain when administered in excessive concentrations. Additionally, mitochondrial damage leads to the liberation of factors that induce apoptosis, resulting in cytoplasmic fragmentation and nuclear condensation; this sufficiently elucidates the aberrant nuclear pattern observed in the groups treated with tramadol across all the age groups analyzed (Kamranian et al. 2023).

GCs with well-defined circular nuclear membranes and clumped chromatin were observed in the current study. The precursors of GCs have the same morphology but are smaller in size. GCs with extensive vacuolations, an irregular nuclear membrane, a dilated Golgi complex and a marked decrease in granule cell precursors are observed in tramadol-treated groups. The observed morphological alterations in the GCL as a result of tramadol treatment in the present study are consistent with previous research indicating that cerebellar granule neurons are particularly susceptible to oxidative stress-related conditions (Kaur et al. 2007; Hassan et al. 2022). Cerebellar granule cell neurons are characterized by their high energy demand and comparatively low ATP levels, in addition to their heightened transcriptional activity of genes linked to oxidative stress and inflammatory responses (Wang et al. 2009). The capacity of these cells to mount effective stress defenses is significantly impaired by these factors, rendering them vulnerable to energy crises in the face of heightened stress (Hao et al. 2023).

TEM micrographs demonstrated that edema of astrocyte processes in close proximity to degenerated PCs was observed in almost all groups treated with tramadol at different stages of postnatal development. The viability of neuronal glial cells is influenced by opioids via a mechanism mediated by opioid receptors, as demonstrated in prior investigations (Pahan and Xie 2023). The expansion of astrocytes, a widely recognized integral response to cytotoxic brain injury, was similarly documented in the current investigation through the upregulation of GFAP-immune-reactive astrocytes. Hyperplasia and hypertrophy of cell bodies and processes are characteristic features of the astrocyte response to injury; these two parameters define reactive gliosis, the most significant impediment to axonal regeneration (Moore and Jessberger 2013). This finding aligns with the observed highly branched hypertrophied astrocyte pattern in GFAP-stained sections following tramadol administration. Specifically, the astrocytes displayed distorted thickened processes and enlarged darkly stained cell bodies (Cikriklar et al. 2016).

In contrast, the present study revealed that the integral components of the blood–brain barrier were substantially affected in tramadol-treated groups; perivascular astrocytes exhibited significantly enlarged processes, and perivascular microglia exhibited significantly increased size. In the tramadol-treated groups, the capillary endothelial cells swelled substantially, resulting in a significant narrowing and eventual occlusion of the capillary lumen. Disrupted endothelial lining and the presence of perivascular microglia in the blood vessel basal lamina may influence the tensile properties of vascular membranes and the biomechanics of individual cells.

In addition, the present study found a substantial increase in serum MDA levels and a significant decrease in serum SOD levels in tramadol-treated groups compared to controls. This may be strongly correlated with degenerative signs and the substantial decrease in oligodendroglia observed in silver-stained sections and disrupted myelin structures detected by TEM in neuronal membranes and axonal nerve endings. The polyunsaturated fatty acid content of neuronal membranes makes them especially susceptible to the oxidative damage caused by free radicals. Therefore, the tramadol-induced oxidative stress effect observed in the present study can adequately explain the neuronal ultrastructural changes and suggests parallel functional impairment, given that effective neuronal functions are essential for specialized conduction and synaptic transmission activity.

Furthermore, the myelin alterations that were noted in the nerve axons during the current investigation may be ascribed to the impact of tramadol on the escalation of lipid peroxidation, protein modifications induced by oxidative stress, and cellular damage mediated by reactive oxygen species (ROS) (Basu and Basu 2020). The present study identified a significant correlation between myelin abnormalities and ultrastructural changes in oligodendrocytes, which are the primary cells responsible for myelin synthesis. Tramadol induces cellular dysfunction in oligodendrocytes; this is well established (Barbosa et al. 2021).

## Conclusion

The administration of tramadol by the mother throughout pregnancy and lactation resulted in detrimental neurotoxic effects on a range of neurons in the cerebellar cortex after birth. Prenatal exposure to tramadol induced structural and oxidative stress-related changes in the cerebellar cortex, accompanied by astrogliosis, according to this study. The current study revealed that tramadol has the ability to elicit detrimental effects on neurons in the cerebellum by means of upregulation of the GFAP and P53 proteins and downregulation of the Ki67 protein. By way of induction of apoptosis, inflammation, oxidative stress, and the release of multiple mediators, these proteins execute their functions. Furthermore, the ingestion of tramadol results in a substantial decrease in the expression of microRNA genes, which has a profound impact on numerous target genes, such as Ak1, p21, and other genes involved in the P53 signaling pathway. Normal regulation of brain size is substantially disrupted when miR7 gene expression is reduced, as the transition from radial glial cells to intermediate progenitor cells is significantly impaired and progenitor apoptosis is increased. In light of this, it is imperative that the appropriate authorities impose significantly stricter limitations on the use of tramadol, especially during pregnancy.

## Data Availability

All data supporting the findings of this study are available within the article. Further enquiries can be directed to the corresponding author.
